# Cysteine peptidases of *Eudiplozoon nipponicum*: a broad repertoire of structurally assorted cathepsins L in contrast to the scarcity of cathepsins B in an invasive species of haematophagous monogenean of common carp

**DOI:** 10.1186/s13071-018-2666-2

**Published:** 2018-03-06

**Authors:** Lucie Jedličková, Hana Dvořáková, Jan Dvořák, Martin Kašný, Lenka Ulrychová, Jiří Vorel, Vojtěch Žárský, Libor Mikeš

**Affiliations:** 1 Department of Parasitology, Faculty of Science, Charles University, Viničná 7, 12844 Prague 2, Czech Republic; 2 Medical Biology Centre, School of Biological Sciences, Queen’s University Belfast, 97 Lisburn Road, Belfast, BT9 7BL UK; 3 Department of Zoology and Fisheries, Faculty of Agrobiology, Food and Natural Resources, Czech University of Life Sciences Prague, Kamýcká 129, 16500 Prague 6, Czech Republic; 4 Department of Botany and Zoology, Faculty of Science, Masaryk University, Kotlářská 2, 611 37 Brno, Czech Republic; 5 Institute of Organic Chemistry and Biochemistry, Academy of Sciences of the Czech Republic, Flemingovo nám. 2, 16000 Prague 6, Czech Republic; 6 Department of Parasitology, Faculty of Science, Charles University, Průmyslová 595, Vestec, 25250 Czech Republic

**Keywords:** Cysteine peptidase, Protease, Cathepsin, S2 subsite, Haematophagy, Blood digestion, Monogenea, Diplozoidae, *Eudiplozoon nipponicum*, Fish parasite

## Abstract

**Background:**

Cysteine peptidases of clan CA, family C1 account for a major part of proteolytic activity in the haematophagous monogenean *Eudiplozoon nipponicum*. The full spectrum of cysteine cathepsins is, however, unknown and their particular biochemical properties, tissue localisation, and involvement in parasite-host relationships are yet to be explored.

**Methods:**

Sequences of cathepsins L and B (*EnCL* and *EnCB*) were mined from *E*. *nipponicum* transcriptome and analysed bioinformatically. Genes encoding two *EnCLs* and one *EnCB* were cloned and recombinant proteins produced in vitro. The enzymes were purified by chromatography and their activity towards selected substrates was characterised. Antibodies and specific RNA probes were employed for localisation of the enzymes/transcripts in tissues of *E. nipponicum* adults.

**Results:**

Transcriptomic analysis revealed a set of ten distinct transcripts that encode *EnCLs*. The enzymes are significantly variable in their active sites, specifically the S2 subsites responsible for interaction with substrates. Some of them display unusual structural features that resemble cathepsins B and S. Two recombinant *EnCLs* had different pH activity profiles against both synthetic and macromolecular substrates, and were able to hydrolyse blood proteins and collagen I. They were localised in the haematin cells of the worm’s digestive tract and in gut lumen. The *EnCB* showed similarity with cathepsin B2 of *Schistosoma mansoni*. It displays molecular features typical of cathepsins B, including an occluding loop responsible for its exopeptidase activity. Although the *EnCB* hydrolysed haemoglobin in vitro, it was localised in the vitelline cells of the parasite and not the digestive tract.

**Conclusions:**

To our knowledge, this study represents the first complex bioinformatic and biochemical characterisation of cysteine peptidases in a monogenean. *Eudiplozoon nipponicum* adults express a variety of CLs, which are the most abundant peptidases in the worms. The properties and localisation of the two heterologously expressed *EnCLs* indicate a central role in the (partially extracellular?) digestion of host blood proteins. High variability of substrate-binding sites in the set of *EnCLs* suggests specific adaptation to a range of biological processes that require proteolysis. Surprisingly, a single cathepsin B is expressed by the parasite and it is not involved in digestion, but probably in vitellogenesis.

**Electronic supplementary material:**

The online version of this article (10.1186/s13071-018-2666-2) contains supplementary material, which is available to authorized users.

## Background

Blood-feeding monogeneans of the family Diplozoidae (Heteronchoinea) are ectoparasites that live on the gills of cyprinid fishes. One member of the family, *Eudiplozoon nipponicum*, is an important invasive species, first recorded in Europe in 1983 on farmed carp in France [1]. It is currently found throughout Europe and is a widespread representative of the helminth fauna of the common carp (*Cyprinus carpio*) in the Czech Republic [2]. The common carp is a fish of high economic importance in many Asian and European countries, with global aquaculture production yielding over 4 million tons in 2014 [3]. Since the parasite affects the health of farmed fish, although precise estimates are unknown, it is assumed it causes economic losses. Pathogenic effects of *E*. *nipponicum* are associated mainly with inducing hypochromic microcytic anaemia in the fish by a continuous intake of blood by worms attached to the carp gills [4].

Monogenea are a rather neglected group of Neodermata and only a handful of papers on their biochemistry and bioactive molecules have ever been published. Based on previous ultrastructural studies, it has been assumed that in diplozoid monogeneans, the digestion of blood, gathered by combined action of their powerful buccal suckers and muscular pharynx, takes place within the lysosomal cycle in the specialised cells of the intestinal epidermis [5–9], similarly as in blood-sucking mites such as ticks [10, 11]. Our previous study [12] has shown that the processing of blood in *E*. *nipponicum* relies on an evolutionarily conserved multi-enzyme network of cysteine and aspartic peptidases, similar to the proteolytic cascades of other blood-feeding helminths such as *Schistosoma*, *Fasciola*, *Ancylostoma*, etc. [13–16]. Among the endopeptidases of *E*. *nipponicum*, clan CA cysteine peptidase activities, including cathepsin L-like and cathepsin B-like activity, are dominant [12].

In helminths, cathepsins L and B play various roles. Due to their histolytic potency, they are involved in host invasion and tissue migration, but they also play a role in various pathological processes, immune evasion, and other parasite-host interactions, as well as in helminth reproduction, nutrient digestion, etc. [17–19]. In general, peptidases encoded in the helminth genomes are mostly temporarily expressed in the various life stages, thus reflecting the parasite’s specific needs regarding digestion or other hydrolytic processes. For instance, a characterisation of several cathepsins L of the liver fluke *Fasciola hepatica* had shown that the infective larvae use cathepsin L3 to traverse the host’s intestinal wall, while the fluke’s migratory stages utilise cathepsin L2 to penetrate host liver tissue, and adults employ cathepsin L1 jointly with cathepsins L2 and L5 to digest host proteins [20]. The use of different peptidases with overlapping substrate specificities helps heteroxenous and tissue-migrating parasites to adapt to various environments and sources of nutrition within the hosts. On the other hand, little is known about the complex functioning of cysteine peptidases in monoxenous blood-feeding monogeneans that spend most of their life attached to a single type of host tissue, such as the gills.

In the present study, we focused on clan CA cysteine peptidases, namely cathepsins L and B, selected due to their abundance in the transcriptome of adult *E*. *nipponicum* worms. We have employed phylogenetic and bioinformatic analysis to investigate their relationship to other helminth peptidases. We have selected two of the most abundant cathepsins L and the only expressed cathepsin B, and produced these as functionally active recombinant forms using the *Pichia pastoris* expression system. A biochemical and functional characterisation of the recombinant enzymes was performed in order to understand their specificity and substrate preference. Furthermore, we have developed specific antibodies and RNA probes to localise proteins/transcripts within the worms’ bodies by immunofluorescence assay and RNA *in situ* hybridisation technique. Our work thus presents the first detailed functional characterisation of monogenean peptidases.

## Methods

### Parasite material

Adult worms of *E. nipponicum* were collected from the gills of infected carps (*C. carpio*) freshly slaughtered in a commercial facility of the fishery Rybářství Třeboň, Plc., Třeboň basin, South Bohemia, Czech Republic. A soluble worm extract, parasite RNA, and first-strand cDNA were obtained as previously described [12]. For RNA *in situ* hybridisation, the worms were flat-fixed between microscopic slides in Bouin’s solution (Sigma-Aldrich, Darmstadt, Germany) at RT for 1 h, then transferred into fresh fixative and incubated at 4 °C overnight. For immunohistochemistry, 4% paraformaldehyde in PBS was employed as a fixative. Fixed worms were dehydrated with increasing concentrations of ethyl alcohol (70–100%), cleared with xylene, and embedded in paraffin.

### Sequence analyses of cathepsin L-like and B-like peptidases of *E. nipponicum*

Transcripts from the transcriptome of adult *E. nipponicum* worms were assembled within a parallel project and deposited in the NCBI GenBank database under accession number GFYM00000000. They were annotated by searching for the closest homologues. We have used BLASTp and BLASTn algorithms (E-value 1e-5) to mine the following public sequence databases: NCBI non-redundant protein database [21], MEROPS database of peptidases and their inhibitors [22], UniProtKB/UniRef100 database and UniProtKB/TrEMBL only used for searching for protein sequences related to phylum Platyhelminthes (Taxon ID: 6157) [23], UniProtKB/Swiss-Prot [24], RCSB PDB [25] and DDBJ [26]. Sequences encoding cathepsin L-like and B-like peptidases of *E*. *nipponicum* were identified and their amino acid sequences (catalytic domains) aligned with sequences of formerly recognised as *E*. *nipponicum* cathepsins L, *EnCL1* (GenBank: KP793605) and *EnCL3* (GenBank: KP793606), *F. hepatica* cathepsin L1 (GenBank: AAT76664.1), *F. hepatica* cathepsin L3 (GenBank: CAC12807.1) and with *E. nipponicum* cathepsin B, *EnCB* (GenBank: MF346929), *Schistosoma mansoni* cathepsins B1 (GenBank: CAD44624.1) and B2 (GenBank: XP_018651608.1), respectively, using Clustal Omega tool (http://www.ebi.ac.uk/Tools/msa/clustalo/) [27]. The relative abundance of cathepsin L transcripts, which reflects the rate of transcription of the corresponding genes, was predicted by back mapping of raw Illumina reads to the assembled transcripts using Kallisto v. 0.43.0 [28]. The presence of a signal sequence in amino acid sequences of the enzymes was predicted by SignalP 4.1 Server (http://www.cbs.dtu.dk/services/SignalP/) [29]. A theoretical position of the pro-region and composition of the enzymes’ S2 subsites of the active site were determined by multiple sequence alignment (see above). Molecular mass and theoretical pI of the deduced proteins were determined by Compute pI/Mw software available at the ExPASy web portal (https://web.expasy.org/compute_pi/) [30]. Potential N-glycosylation and O-glycosylation sites were identified using an online tool at NetNGlyc 1.0 and NetOGlyc 4.0 Servers (http://www.cbs.dtu.dk/services/NetNGlyc [30] and http://www.cbs.dtu.dk/services/NetOGlyc [31]).

*Eudiplozoon nipponicum* cathepsin L1 and L2 amino acid sequences were used as queries for BLAST searches in the NCBI non-redundant protein database [32] and the Wormbase database of platyhelminths [33]. Recovered sequences were further analysed using CLANS software [34] and a cluster of 515 sequences containing *E. nipponicum* cathepsins was selected. Redundant sequences were filtered at 95% level of identity using the CD-HIT software [35]. Divergent and incomplete sequences were manually removed, which resulted in a dataset of 288 sequences. These were aligned using the MAFFT [36] and the alignment was trimmed using BMGE (239 sites) [37]. We inferred a maximum-likelihood tree using the best-fit model (LG + C60 + G), and tested the topology with 10,000 ultrafast bootstraps in IQ-TREE [38]. Bayesian inference of phylogeny was run under the C60 site-heterogeneous mixture model using Phylobayes.

### Expression of recombinant cathepsins L1, L3 and B in *Pichia pastoris* and their purification

Two abundant *EnCLs* (*EnCL1* and *EnCL3*) and the only *EnCB* were selected for a functional expression. Gene-specific primers (Additional file 1) and the first-strand cDNA of adult *E. nipponicum* were used to amplify the *yrEnCL1* (GenBank: KP793605), *yrEnCL3* (GenBank: KP793606), and *yrEnCB* (GenBank: MF346929) genes. The amplified products were ligated into a *P. pastoris* expression vector pPICZα B as described previously [39], and the constructs were verified by Sanger sequencing. Protein expression in *P. pastoris* X-33 was carried out following a protocol described previously [40] and according to the manual of the Easy Select Pichia Expression Kit (Thermo Fisher Scientific, Waltham, Massachusetts). The yeast medium was centrifuged and the supernatant filtered (0.22 μm) and concentrated on AmiconUltra-15 filters 10,000 MWCO (Millipore, Darmstadt, Germany). The enzymes were affinity purified via their His-tag using Ni-chelating chromatography (HisTrap™ FF crude, GE Healthcare, Little Chalfont, United Kingdom) and a stepwise elution by imidazole (0.05–0.5 M). Yeast-origin hyperglycosylation of *EnCB* was removed by endoglycosidase F1 (Calbiochem, Darmstadt, Germany). Purification was completed by cation exchange FPLC on Mono S™ 5/50 GL column (GE Healthcare) using 20 mM MES buffer pH 6.0 and gradient elution by NaCl (0–1 M).

### Expression of recombinant cathepsin L1 in *Escherichia coli* and its purification

The *EnCL1* gene (GenBank: KP793605) was amplified using first-strand cDNA of adult *E. nipponicum* and specific primers (Additional file 2). The amplified product was ligated into the *E. coli* expression vector pet28a + (Novagen, Darmstadt, Germany) and verified by DNA sequencing. *EnCL1* was expressed in *E. coli* BL21 Star™ (DE3) (Invitrogen, Carlsbad, California) according to pET System Manual (Novagen). Harvested cells were lysed in 20 mM Tris-HCl pH 8/ 0.3 M NaCl/1% lauryl sarcosine/10 mM imidazole by sonication, and centrifuged at 10,000× *g* for 10 min. Inclusion bodies were solubilised with 6 M guanidine hydrochloride as described elsewhere [41]. The solubilised mixture was filtered (0.65 μm), passed over the column containing Ni-NTA beads (Qiagen, Hilden, Germany), and eluted by imidazole (0.05–0.5 M). Chromatographic fractions were analysed by SDS-PAGE and the identity of suspected protein bands was verified by mass spectrometry as described in [12].

### Processing the *yrEnCL3* pro-enzyme

Auto-processing of *pro-yrEnCL3* was tested by incubation of the recombinant protein in 50 mM/100 mM CPB, with 2 mM DTT at pH values of 4, 5 and 6. Aliquots of 20 μl were taken from reactions at specific time intervals (15, 30, 60 and 120 min) at 37 °C, whereby incubations were stopped by adding 10 μM E-64. Processing of the enzyme to its mature form was monitored by SDS-PAGE, Western blotting and Edman degradation of N-terminus and subsequently confirmed by fluorometry with the corresponding synthetic peptide substrates (see below). Processing of *yrEnCL3* was also monitored by affinity labelling using 20 μM fluorescent probe BODIPYgreen-DCG-04 [42] and 2.5 μg of the enzyme (30 min incubation at pH 5) as described previously [40]. Specificity of the probe towards the active site of the peptidase was verified by pre-incubation with the cysteine peptidase-specific inhibitor E-64 (10 μM) for 5 min.

### Peptidase activity assays

Peptidase activities of recombinant cathepsins were measured with fluorogenic aminomethylcoumarin peptide substrates (Bachem) as described previously [12]. Substrates tested with *yrEnCL1* and *yrEnCL3* included Z-Phe-Arg-AMC (FR), Z-Leu-Arg-AMC (LR), Z-Arg-Arg-AMC (RR), Z-Pro-Arg-AMC (PR) and Z-Gly-Pro-Arg-AMC (GPR). The *yrEnCB* was tested with FR and RR substrates only. The purified *rEnCLs* (15 nM) and *rEnCB* (30 nM) were dissolved in 100 μl of 50 mM/100 mM CPB supplemented with 2 mM DTT, in pH range of 3–8. The reactions were initiated by adding the individual substrates (final concentration 50 μM) in 100 μl of the same buffer at 28 °C. The release of free AMC was measured at excitation and emission wavelengths of 355 and 460 nm, respectively, in Infinite M200 fluorometer (TECAN, Männedorf, Switzerland).

For inhibition assays, three inhibitors were used (final concentration 10 μM, Sigma-Aldrich): E-64 (L-trans-epoxysuccinyl-leucylamido [4-guanidino] butane), which is a general cysteine peptidase inhibitor, iCL (Arg-Lys-Leu-Leu-Trp-NH2), a reversible inhibitor of cathepsin L; and CA-074 (N-[L-3-trans-propylcarbamoyloxirane-2-carbonyl]-Ile-Pro-OH), which is a selective inhibitor of cathepsin B. Inhibitors were mixed with enzyme samples and incubated at 28 °C for 15 min prior to adding the substrates. Remaining hydrolysis rates were measured with the FR substrate (cathepsins L and B), with LR (cathepsins L), or with RR (cathepsin B) as described above.

The assay of exopeptidase activity of activated *yrEnCB* (75 nM) was carried out with benzoyl-glycinyl-histidinyl-leucine (Bz-Gly-His-Leu; final conc. 2 mm) as a substrate, using a modified protocol [43]. The activity was measured in 50 mM/100 mM CPB pH 4–6 containing 2 mM DTT and 0.05 mg/ml fluorescamine.

### Hydrolysis of macromolecular substrates by recombinant cathepsins L and B

Processed *yrEnCL1*, *yrEnCL3* and *yrEnCB* (2.5 μg each) were incubated at 37 °C with 50 μg of macromolecular substrates (bovine haemoglobin and albumin, human collagen I, IgG and fibrinogen; Sigma-Aldrich) dissolved in 100 μl of 50 mM/100 mM CPB with 2 mM DTT in pH 4.5–6. Aliquots (10 μl) for SDS-PAGE analysis in 12% gels were taken at various intervals (0, 30, 60 and 120 min and 16 h).

### RNA *in situ* hybridisation

*EnCL1*/*EnCL3* gene-specific primers (Additional file 3) and first-strand cDNA of adult *E. nipponicum* were used for the PCR. The amplified products were ligated into pGEM®-T Easy vector (Promega, Madison, Wisconsin) and verified by DNA sequencing. The constructs were linearised and used as a template for RNA probes. Both sense and anti-sense RNA probes were synthesised in vitro (Dig RNA Labelling Kit (SP6/T7); Roche, Basel, Switzerland). *In situ* hybridisation was performed using a modified protocol [44]. Briefly, the sections of adult worms on slides were incubated for 19 h at 41 °C (*EnCL1*) and at 37 °C (*EnCL3*) with specific RNA probes diluted 1:100 in a hybridisation mixture (5× SSC, 1× PBS, 50% deionised formamide, 1% Tween-20, 10% dextran sulphate Mw 500×, 1 mg/ml Torula yeast RNA). Detection was achieved with alkaline phosphatase-conjugated anti-digoxigenin antibodies (1:500, Roche) and Fast Red TR substrate (Sigma-Aldrich). Negative controls were incubated under the same conditions but with an anti-sense probe or without a probe. Specific strand RT-PCR was used for the detection of natural anti-sense transcripts occurring in the monogenean cells [45].

### Production of peptidase-specific polyclonal antibodies

For the production of monospecific polyclonal *anti-EnCL3* and *anti-EnCB* antibodies, ICR/CD1 mice (ENVIGO, Huntingdon, United Kingdom) were injected subcutaneously twice, at a 14-day interval, with 30 μg of purified *pro-yrEnCL3* or deglycosylated *yrEnCB* in sterile saline and TitermaxGold adjuvant (Sigma-Aldrich). The mice were boosted by an intramuscular injection of purified antigens (15 μg) in sterile saline after another two weeks. *Anti-EnCL1* antibodies were produced in mice injected intraperitoneally with *c.*50 μg of *brEnCL1* cut as a band from a 12% gel after SDS-PAGE. The strip of the gel containing the antigen was repeatedly and thoroughly washed in 50% methanol/10% acetic acid for several hours to remove SDS, washed several times in sterile saline, and homogenised in sterile saline using a motorised pellet pestle. Two antigen deliveries were performed at an interval of 14 days. All mice were bled under deep ketamine/xylazine anaesthesia 14 days after the last injection and sera were collected by centrifugation. Control sera were taken from the same mice prior to immunisation.

### Western and affinity blotting

Specificity of the anti-cathepsin antibodies produced in mice was verified on PVDF membrane immunoblots of soluble worm extracts previously separated by SDS-PAGE in 12% gels (20 μg/well). The experiment was performed according to a modified protocol [40]. The membranes were first incubated for 1 h with mouse *anti-EnCL3/anti-EnCL1/anti-EnCB* /control sera diluted 1:100 in PBS-T and then for 1 h with horseradish peroxidase-labelled goat anti-mouse IgG (Sigma-Aldrich) diluted 1:5000 in PBS-T. Finally, the membranes were developed by the Opti-4CN Substrate Kit (Bio-Rad, Hercules, California).

Purified recombinant proteins (1 μg/well) were resolved by SDS-PAGE in 12% gels and either stained by CBB or trans-blotted onto a PVDF membrane. His-tagged enzymes were detected on blots either with a mouse biotinylated monoclonal Anti-polyhistidine antibody (Sigma-Aldrich), alternatively with 5 nM iBody4, which is a biotinylated copolymer containing nitrilotriacetic acid-bound nickel cations [46]. In both cases, detection was finalized using peroxidase-labelled streptavidin (Sigma-Aldrich) and the Opti-4CN™ Substrate kit.

### Immunohistochemistry

Deparaffined and rehydrated 5 μm sections of worms on slides were heated 3 × 3 min in 0.01 M citrate buffer pH 6 containing 0.05% Tween 20 in a microwave oven (500 W) and then allowed to cool for 20 min. Subsequently, the sections were blocked and immunostained according to a modified Immunocytochemistry and Immunofluorescence Protocol (Abcam). After an overnight incubation with sera (1:50), the slides were placed in a dark chamber for 1 h at RT with Goat Anti-Mouse IgG H&L-Alexa Fluor® 568 (Ex: 578 nm/Em: 603 nm, Abcam, Cambridge, United Kingdom) diluted 1:200 in PBS-TX100 with 1% BSA. Finally, the sections were mounted in Vectashield with DAPI (Vector laboratories, Burlingame, California). Immunofluorescence was observed and photographed under fluorescence or confocal microscope.

## Results

### Bioinformatic and phylogenetic analyses of the primary structures of the enzymes

Ten unique transcripts encoding different cathepsin L-like sequences were discovered in the transcriptome of adult *E. nipponicum.* Of the predicted CL amino acid sequences, seven represented complete sequences of mature (processed) enzymes, with six encoding the complete sequences of zymogens including the pro-sequences. Two of the predicted complete zymogen sequences were identical with the sequence of *EnCL1* (GenBank: KP793605) and *EnCL3* (GenBank: KP793606) obtained previously by reverse transcription of parasite mRNA using degenerate primers, followed by RACE-PCR [12]. The complete pro-enzyme molecules were assigned *EnCL1-EnCL6*. The remaining four incomplete sequences shared a relatively high similarity with *EnCL6*: the five close orthologs were therefore assigned *EnCL6a-EnCL6e*. The alignment of the predicted amino acid sequences of mature *EnCLs* with cathepsins L1 (GenBank: AAT76664.1) and L3 (GenBank: CAC12807.1) of *F. hepatica* is presented in Additional file 4, including a mutual comparison of the amino acids of the S2 pocket, which determines the substrate-binding specificity of the enzymes. The alignment of all complete/incomplete amino acid sequences of *EnCLs* is included in Additional file 5.

Interestingly, *EnCL2* displays a substitution of the active site cysteine (Cys25) to serine (Ser25). In terms of amino acid constitutions of the S2 pocket (Additional files 4, 5 and 6), the *EnCLs* can be divided in 3 groups. One is formed by CL1, CL3, CL4 and CL5, which share the Leu67, Ala133, Leu157 motif (papain numbering used throughout this column), with a slightly hydrophobic Ala being at the bottom of the S2 pocket. The second includes *EnCL6a*, *EnCL6b* and *EnCL6e*, where Ala133 is replaced by the amphiphilic Gly133 (for *EnCL6c* and *EnCL6d* data were not available.). The third group contains only *EnCL2*, in which the S2 pocket differs substantially from that of the other *EnCLs*: Ala133 is replaced by the more hydrophobic Val133 at the bottom, and moreover, the two leucines are replaced by Trp67 and Phe157, which could make the S2 pocket highly hydrophobic. The most variable element in the S2 pocket is the amino acid position 205, where hydrophobic residues occur in CL3, CL4 and CL5, and uncharged hydrophilic residues in CL1 and CL6a. In the case of CL2, residue 205 is a positively charged Arg, whereas in CL6e, it is the negatively charged aspartate.

Some of the *EnCLs* include potential N-glycosylation sites: CL2 has one and CL4 has two in the mature enzyme, others occur in the pro-sequences of CL2, CL4 and CL5. Several O-glycosylation sites have been predicted in all but one (*EnCL6b*) cathepsin L sequences. Most, however, are located in the pro-domains; CL5 has three, while all the other enzymes except for CL6d have one in the mature part (Table 1, Additional file 5).

**Table 1:** Predicted/computed sequence-derived features of *E*. *nipponicum* cathepsins L and B

	Transcript_ID	Bases/AA	Completeness	SP	pI proenzyme/mature enzyme	MW proenzyme/mature enzyme (kDa)	N-/O-glyc sites
CL1	E_nip_trans_58808_m.372114	954/317	yes	no	5.86/6.08	34.9/24.36	0/7
CL2	E_nip_trans_70234_m.461805	1029/342	yes	yes	7.23/4.87	37/24.9	3/2
CL3	E_nip_trans_02967_m.11341	1107/368	yes	yes	4.80/4.15	38/24.1	0/5
CL4	E_nip_trans_06099_m.25531	1062/353	yes	yes	5.59/4.94	37.6/24.2	3/8
CL5	E_nip_trans_65378_m.396731	1071/356	yes	no	5.62/5.02	39.5/24	1/8
CL6a	E_nip_trans_15113_m.115989	1059/352	yes	yes	5.55/4.8	37.7/24.4	0/2
CL6b	E_nip_trans_04751_m.20488	519/173	iN	?	?/?	?/?	0/0
CL6c	E_nip_trans_04670_m.20209	642/214	iC	yes	?/?	?/?	0/4
CL6d	E_nip_trans_55822_m.362291	705/235	iC	yes	?/?	?/?	0/1
CL6e	E_nip_trans_60687_m.380367	768/255	iN	?	?/4.58	?/24.47	0/2
CB	E_nip_trans_02724_m.9562	1149/382	yes	yes	7.21/5.55	39.9/28.5	1/7

*Abbreviations*: *AA* Amino acids, *SP* Signal peptide, *N-/O-glyc sites* Number of potential N-glycosylation/O-glycosylation sites, iN Incomplete N-terminus, *iC* Incomplete C-terminus, *?* data not available due to an incomplete sequence

**Fig. 1:**
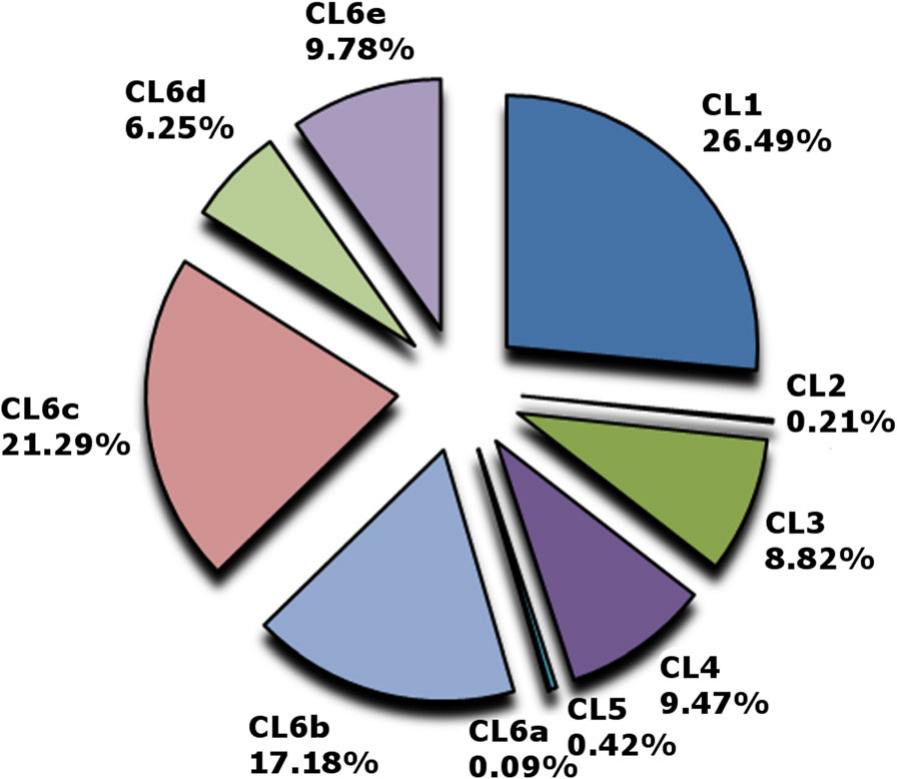
Relative transcription rate of *E*. *nipponicum* cathepsins L. The relative transcription rate predicted by a back mapping of raw Illumina reads to the assembled transcripts was calculated as the percentage of all cathepsin L transcripts

The rate of transcription of the particular *EnCLs* was predicted by a reverse mapping of raw reads towards the selected transcripts. *EnCL1* is the most abundant and *EnCL3* is the sixth most abundant cathepsin L in *E*. *nipponicum* adults (Fig. 1).

**Fig. 2:**
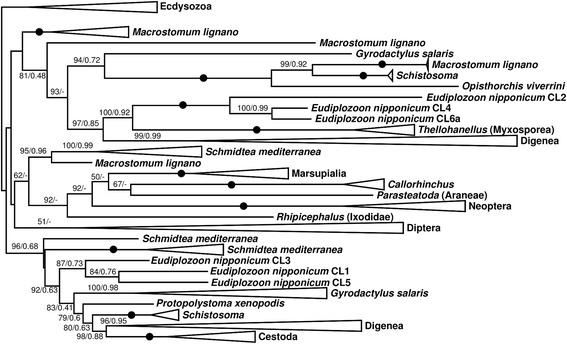
Collapsed phylogram showing relationships between *E. nipponicum* cathepsins L and cathepsins L of other selected organisms. Collapsed unrooted maximum-likelihood tree of selected *E. nipponicum* cathepsins L inferred using the best-fit model (LG + C60 + G). Leaves of related organisms are collapsed. Ultrafast bootstrap supports and posterior probabilities are shown where ultrafast bootstrap is ≥ 50 or posterior probability ≥ 0.9. Maximum support (100/1.0) is indicated by a black circle

Phylogenetic analysis has shown that *E*. *nipponicum* cathepsins L separate into two clusters: one contains CL1, CL3 and CL5, and the other includes CL2, CL4 and CL6a. The two clusters belong to separate clades, whereby each of the clusters also includes different cathepsins L of free-living rhabditophorans and CLs of digeneans and cestodes (Fig. 2, Additional file 7). It can thus be concluded that *E*. *nipponicum* cathepsins L are polyphyletic.

The cluster containing *EnCL1* and *EnCL3*, for which the function in blood digestion was confirmed in this study, occurs within the same clade as the CL of another haematophagous monogenean, *Protopolystoma xenopodis*. The various CLs of the mucophagous *Gyrodactylus salaris* fall into both clades where the two clusters of *EnCLs* are located. Interesting is the highly supported position of myxosporean CLs within the neodermatan clade which contains also *EnCL2*, *EnCL4* and *EnCL6a*.

In stark contrast to the numerous *EnCLs*, only one transcript encoding *EnCB* was found in the *E*. *nipponicum* transcriptome; it contained the whole sequence of the pro-enzyme. Its closest relative found in the databases is cathepsin B2 of *Schistosoma mansoni* (GenBank: XP_018651608.1) (see the alignment in Additional file 8). Its primary structure contains the typical motif of an occluding loop, which is responsible for peptidyl dipeptidase activity of cathepsins B. One potential N-glycosylation site is positioned on the occluding loop. Seven O-glycosylation motifs were predicted in the molecule, five of them located within the pro-domain. *EnCB* also contains a modified ‘haemoglobinase motif’ (213)YWLIA**N**SW--EWGD(226) in the asparagine active site region, which is ascribed to haemoglobinolytic cathepsins B of helminth blood-feeders [47]. The predicted/computed basic features of all *EnCLs* and *EnCB* are summarised in Table 1.

### Functional expression and purification of recombinant cathepsins L1, L3 and B

**Fig. 3:**
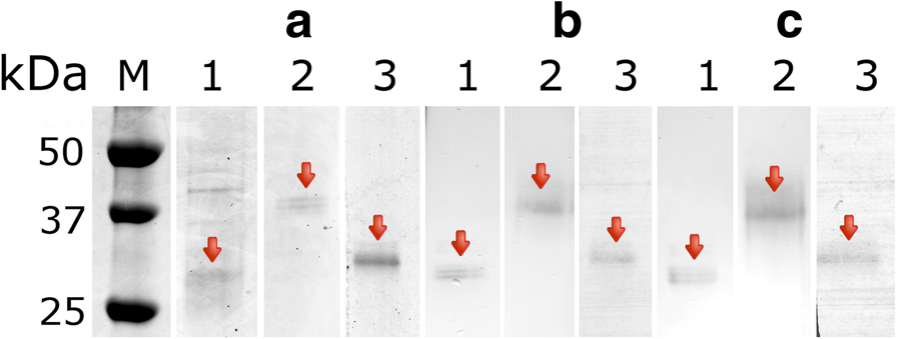
SDS-PAGE and affinity−/immunoblots of *yrEnCL1*, *yrEnCL3* and *yrEnCB* after purification. **a** 12% gel stained with Coomassie Brilliant Blue. **b** affinity blot labelling by iBody4. **c** immunoblot detection by anti-His-Tag antibody. Lane 1: *yrEnCL1* (28 kDa partially processed form); Lane 2: *yrEnCL3* (38 kDa pro-enzyme); Lane 3: *yrEnCB* (29 kDa deglycosylated processed enzyme). Arrows point to the enzyme bands

*EnCL1*, *EnCL3* and *EnCB* were expressed as pro-enzymes in yeasts. Since *yrEnCL1* and *yrEnCB* had undergone self-processing already in yeast cultivation medium, a high activity of both was confirmed in the presence of appropriate fluorogenic peptide substrates. The *yrEnCL1* thus migrated in electrophoretic gels as a ~28 kDa dominant double band and reacted on the blot with anti-His-Tag mouse antibody and iBody4 at the same size (Fig. 3). The enzyme was partially purified by Ni-NTA affinity chromatography, but attempts to purify it to homogeneity by ion-exchange chromatography failed due to its poor stability. Activity and inhibition assays were therefore performed with only partially purified enzyme. Additionally, for the production of anti-*EnCL1* antibodies it was necessary to use the inactive recombinant form produced in *E. coli* (*brEnCL1*).

**Fig. 4:**
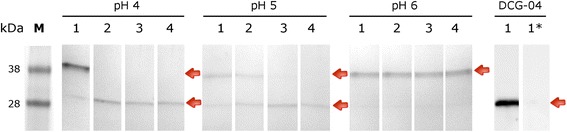
SDS-PAGE and active site labelling of purified *pro-yrEnCL3* after auto-activation at various pH. The 38 kDa zymogen was autocatalytically processed to a 28 kDa form during incubation in 50 mM/100 mM CPB containing 2 mM DTT at 37 °C. After incubation at various pH values, the protein was run in 12% polyacrylamide gels and stained with Coomassie Brilliant Blue (pH 4, pH 5 and pH 6). Lane 1: 15 min incubation; Lane 2: 30 min; Lane 3: 60 min; Lane 4120 min. DCG-04, a 12% gel showing active site labelling of the processed enzyme with fluorescent affinity probe BODIPY green DCG-04. Lane 1: labelling by the affinity probe after 30 min incubation at pH 5; 1*, binding of the affinity probe was blocked by E-64 inhibitor. The 38 kDa zymogen and the 28 kDa processed enzyme are indicated by arrows

The affinity purified unprocessed *pro-yrEnCL3* migrated in the gel as a double band at ~38 kDa, and reacted with the anti-His-Tag antibody and iBody4 at the same size (Fig. 3). Autocatalytic processing of *pro-yrEnCL3* was most effective at pH 4, and resulted in a ~28 kDa band in the gel. Processing of the zymogen was less effective at pH 5, while at pH 6 it did not take place even after prolonged incubation. Affinity probe BODIPYgreen-DCG-04 bound strictly to the mature enzyme, while in the pro-enzyme no labelling was observed (Fig. 4). Edman degradation revealed LPTDVD sequence at the N-terminus of the processed *EnCL3*. Since the processed enzyme had limited stability, the purified pro-enzyme was used for the immunisation of mice.

*YrEnCB* pre-purified on a Ni-NTA column migrated as a broad band around 50 kDa. After enzymatic deglycosylation and subsequent purification by ion-exchange chromatography, it migrated as a ~29 kDa band in the gels, which correlated with the results of Western and affinity blotting (anti-His-Tag antibody, iBody4) (Fig. 3).

### Activity and inhibition assays

**Fig. 5:**
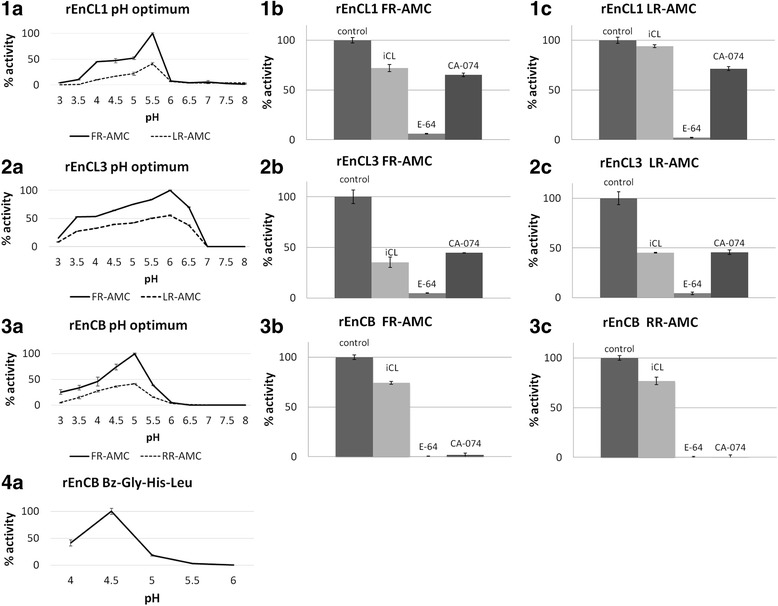
pH optima of *rEnCLs* and *rEnCB* activities, and the effect of inhibitors on enzyme activities. Activity assays were performed in 50 mM/100 mM CPB with 2 mM DTT at pH 3–8. Inhibition assays were run at the pH optima of the particular enzymes. The values are expressed as the percentage of maximum activity in the sample without inhibitor. **1**, *yrEnCL1*; **2**, *yrEnCL3*; **3**, *yrEnCB*; **4**, *yrEnCB*. **a** pH optima of endopeptidase (1–3) and exopeptidase (4) activities. **b** inhibition assays with FR substrate. **c** inhibition assays with LR substrate (1–2) and RR substrate (3). The inhibitors (10 μM) are indicated in the graphs above the columns: E-64, general irreversible inhibitor of clan CA cysteine peptidases; iCL, reversible peptide inhibitor of cathepsin L; CA-074, irreversible inhibitor of cathepsin B

Both *EnCL*s efficiently hydrolysed the FR substrate, and to a lesser extent the LR. No activity towards RR, PR and GPR was detected. The optima for hydrolysis of both substrates were pH 5.5 for *yrEnCL1* and pH 6 for *yrEnCL3*. The preferred substrate for *yrEnCB* was the FR, while RR was cleaved less efficiently at the optimum of pH 5 (Fig. 5).

Activity of all three enzymes was effectively inhibited by 10 μM E-64 (the general inhibitor of clan CA family C1 cysteine peptidases) in the presence of either substrate. The cathepsin B-selective inhibitor CA-074 had completely suppressed the activity of *yrEnCB* at 10 μM and diminished the activity of *yrEnCL1* and *yrEnCL3* to ~70% and 45–65%, respectively, depending on the substrate. The reversible peptide inhibitor of cathepsin L (10 μM, iCL) partially inhibited all three cathepsins but was most effective in the case of *yrEnCL3* with both substrates (*c.*40% remaining activity). The inhibition rate of *yrEnCL1* was comparable with the effect on *yrEnCB* (*c.*90–70% remaining activity) (Fig. 5).

The exopeptidase (dipeptidyl peptidase) activity of *yrEnCB* peaked at pH 4.5, then rapidly dropping to *c.*40% at pH 4, and to less than 20% at pH 5 (Fig. 5).

### Hydrolysis of macromolecular substrates by recombinant cathepsins L and B

**Fig. 6:**
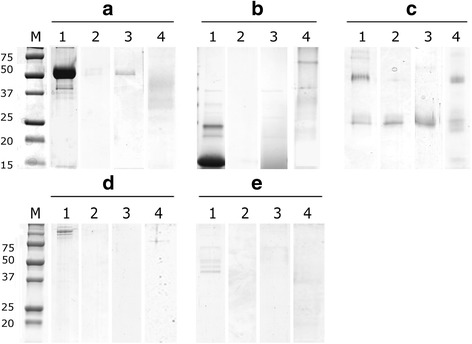
SDS-PAGE of protein substrates degraded by recombinant *EnCL1*, *EnCL3* and *EnCB*. Prior to electrophoresis in 12% polyacrylamide gel, the enzymes were incubated overnight with various protein substrates in 50 mM/100 mM CPB pH 4.5 containing 2 mM DTT. **a** Bovine serum albumin. **b** Bovine haemoglobin. **c** Human IgG. **d** Human type I collagen. **e** Human fibrinogen. Lane 1: controls (substrates without enzyme); Lane 2: *yrEnCL1*; Lane 3: *yrEnCL3*; 4: *yrEnCB*. Gels were stained with Coomassie Brilliant Blue

Both *EnCLs* and *EnCB* efficiently degraded selected protein substrates (bovine haemoglobin and albumin, human collagen type I and fibrinogen) at pH 4.5. *EnCLs* readily hydrolysed also human IgG, which was poorly cleaved by *yrEnCB* at pH range 4.5–6.0 (Fig. 6). While the action of *yrEnCL1* did not vary substantially between pH 4.5–6.0, *yrEnCL3* did not act on the substrates (except fibrinogen) at pH 5.0 or higher (not shown). At pH, hydrolysis of all the macromolecular substrates by *yrEnCB* failed (not shown).

### Western blotting and immunohistochemistry

**Fig. 7:**
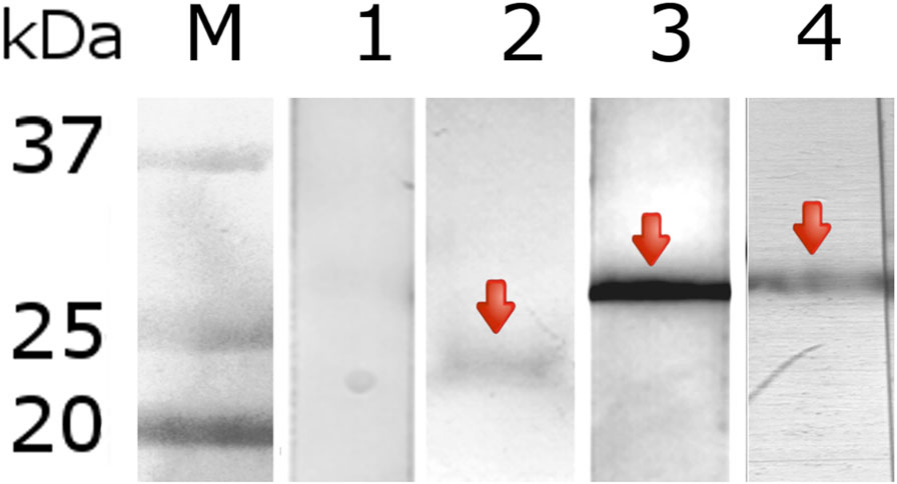
Reactions of anti-cathepsin antibodies with proteins on blots of *E. nipponicum* soluble extracts. Lane 1: mixed control (pre-immune sera); Lane 2: *anti-brEnCL1* antibodies; Lane 3: *anti-pro-yrEnCL3*; Lane 4: *anti-yrEnCB*. Arrows point to the enzymes detected by specific antibodies

Specificity of mouse *anti-EnCL1*, *anti-EnCL3*, and *anti-EnCB* antibodies was verified on immunoblots of the soluble worm extracts. The detected bands appeared at ~24 kDa (*EnCL1*), ~28 kDa (*EnCL3*) and ~29 kDa (*EnCB*). Reactions of the particular sera with the appropriate enzyme bands were highly specific (Fig. 7), so they could be used for immunohistochemistry. Control pre-immune sera did not exhibit any reaction.

**Fig. 8:**
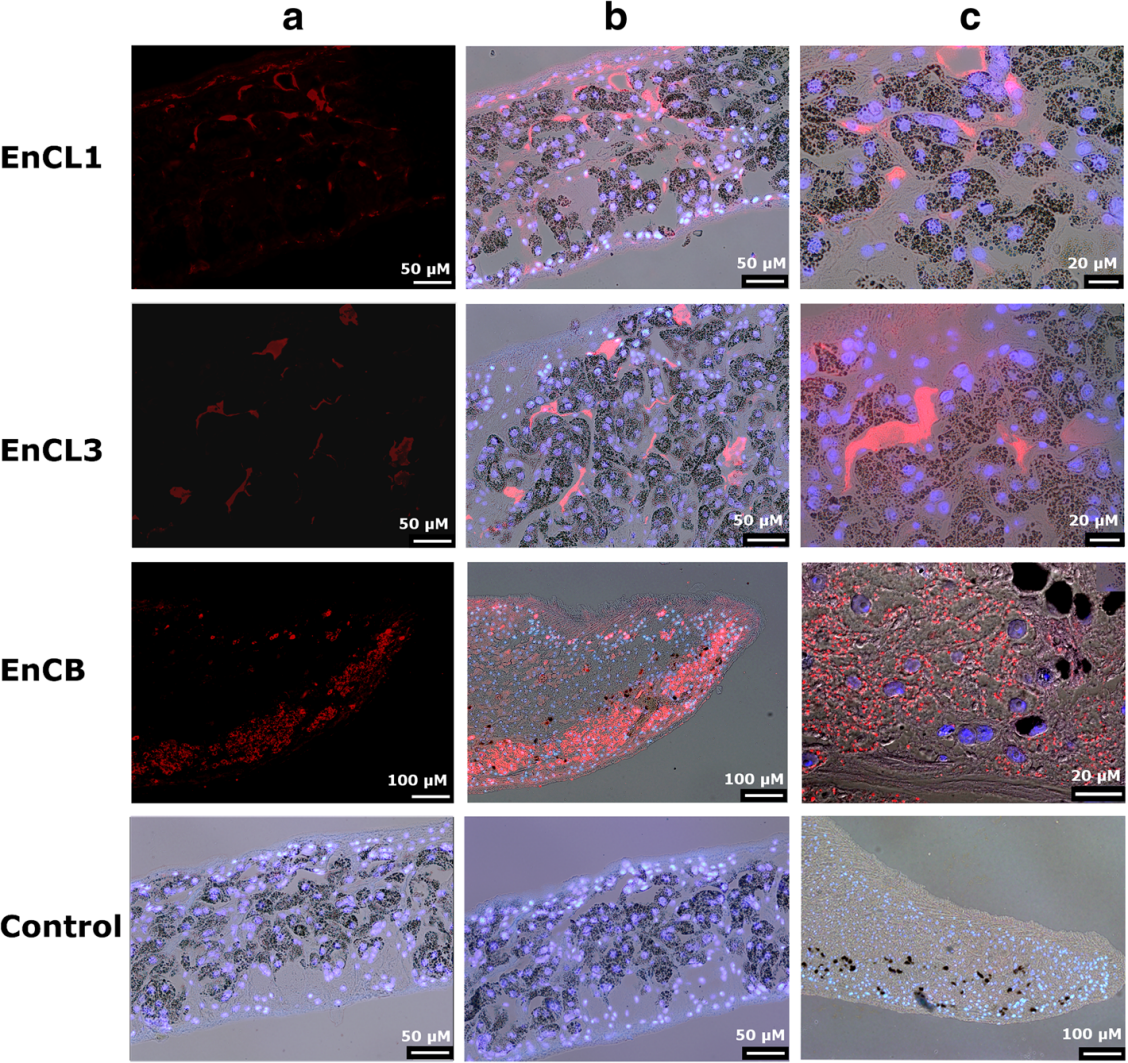
Immunolocalisation of *EnCL1*, *EnCL3*, and *EnCB* on histological sections of adult worms. Longitudinal sections of *E. nipponicum* adult; anterior part of the worm’s body is shown. *EnCL1*, reaction with *anti-brEnCL1* antibodies; *EnCL3*, *anti-pro-yrEnCL3* antibodies; *EnCB*, *anti-yrEnCB* antibodies. **a** Red fluorescence (Alexa Fluor 568 goat anti-mouse IgG) of the labelled secondary antibody in a dark field. **b** Merged picture of bright field view, red antibody fluorescence, and blue DAPI fluorescence. **c** Higher magnification of **b**. Reaction of antibodies raised against *brEnCL1* and *yrEnCL3* takes place in the lumen of the gut. Reaction of antibodies raised against *yrEnCB* takes place in the vesicular structures within vitelline cells. Control, pre-immune mouse sera in the order *EnCL1* (**a)**, *EnCL3* (**b**), *EnCB* (**c**); merged picture of bright field view, red and blue fluorescence. No reaction with any of the control sera was detected

The reactions of specific antibodies with histological sections of the worms showed the presence of both *EnCL1* and *EnCL3* in the lumen of the gut (Fig. 8). No reaction was observed in the haematin digestive cells or outside the digestive system.

*Anti-yrEnCB* antibodies reacted with vesicular structures in the vitelline cells of the adult parasites (Fig. 8). All control sera displayed no reaction.

### RNA *in situ* hybridisation

**Fig. 9:**
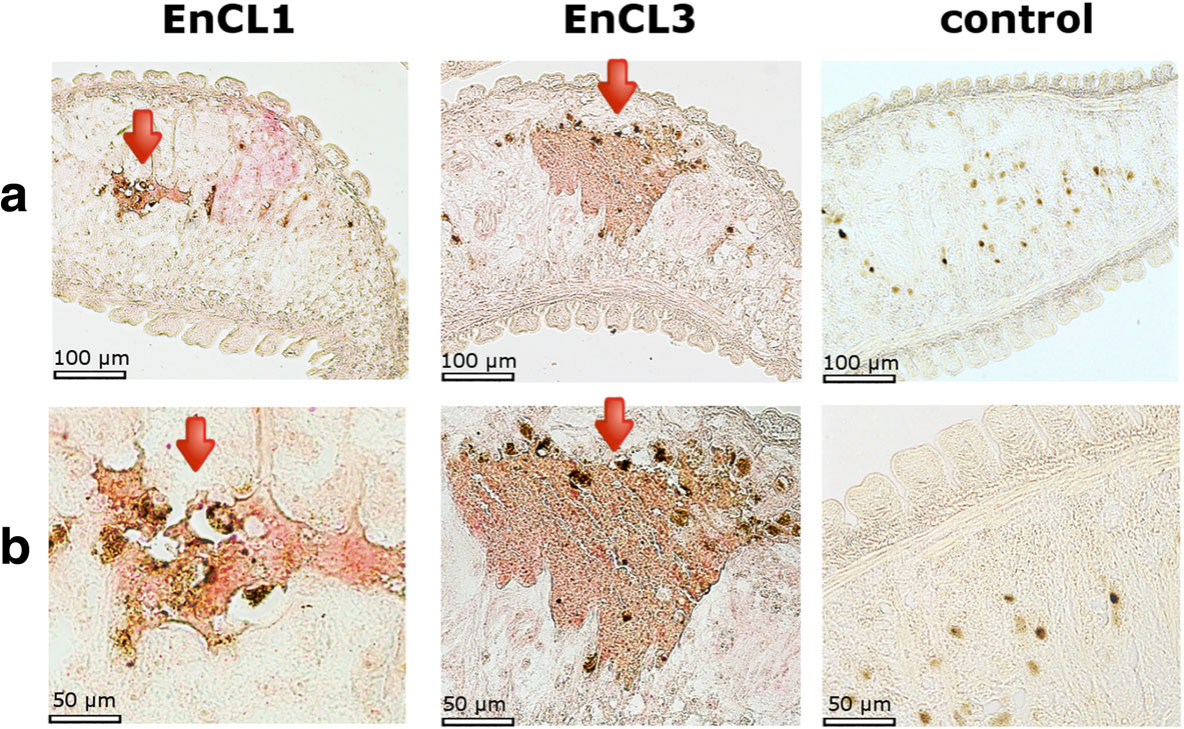
Localisation of mRNA encoding *EnCL1* and *EnCL3* using RNA *in situ* hybridisation. Longitudinal sections of *E. nipponicum* adult worms incubated with specific *EnCL1*/*EnCL3* antisense-RNA probes. **a** Reaction in haematin cells of the digestive tract. **b** Higher magnification of the reacting areas from the same section. Controls were performed without any probe because the worms also produce anti-sense transcripts

*In situ* hybridisation with specific antisense-RNA probes showed a localisation of *EnCL1* and *EnCL3* transcripts specifically in the haematin (digestive) cells of the worms’ gastrodermis (Fig. 9). No reactivity was found in other parts of the parasite body, including tegument, vitelline cells and the initial part of the digestive tract. The same pattern of reactivity was obtained when the sense-RNA probes were used. This was not a non-specific reaction, as demonstrated by the strand-specific RT-PCR which confirmed the presence of anti-sense transcripts in *E*. *nipponicum*. In the negative controls (without any probe), no reaction was observed.

## Discussion

In a previous study [12], we proposed that cathepsin L-like peptidases, in cooperation with cathepsins D and B, play a leading role in the digestion of haemoglobin in the sanguinivorous monogenean *E. nipponicum*. Although the presence of cathepsins L in soluble extracts of the worms was suggested by several methods in that study, the large number of different cathepsin L transcripts (10 in total) found in the transcriptome of the adult life stage was not anticipated. In other blood-feeding helminths, such as *Fasciola hepatica*, multiple cathepsins L are involved in feeding and other important tasks, such as tissue migration, immune evasion, and possibly egg production and metacercarial excystment [48, 49]. Small changes (even single amino acid substitutions) in the primary structure, but especially in the S2 subsite of the active site of those cathepsin L types/isoforms, alter the substrate specificity [50–52]. The production of peptidases with overlapping specificities enables the parasite to degrade host-derived macromolecules more efficiently.

All but one of the *EnCLs* characterised in this study possess a typical catalytic triad of cysteine peptidase active site (Cys/His/Asn); the exception is *EnCL2*, in which the active site cysteine (Cys25) is replaced by serine (Ser25). However, as described for congopain (cathepsin L-like peptidase) in *Trypanosoma congolense*, Cys-to-Ser mutation need not necessarily affect the proteolytic ability of an enzyme [53]. It has been also suggested that Cys-to-Ser mutation in the cysteine peptidases of parasites might be a way to moderate oxidation of the active site in case parasites have to deal with an oxygen-rich environment, such as blood or external environment [17]. In addition to this substitution in the active site, *EnCL2* has in its S2 pocket Trp at position 67 (papain numbering used throughout the Discussion), which makes it a potential collagenase/elastase. Presence of the bulky aromatic residues Tyr67/Trp67 is typical of *F. hepatica* cathepsins L2 and L3, and of human cathepsin K, which all efficiently cleave collagen and elastin of the connective tissues due to their ability to accommodate Pro residues at P2 position in the substrates via a stabilising interaction with Tyr67/Trp67 [50–52, 54, 55]. However, the functionality and possible role of *EnCL2* has not yet been studied. Its expression rate in adult worms is very low, which indicates that the enzyme is predominantly employed by another life stage (the larva: oncomiracidium; or the post-oncomiracidial stage: diporpa), or in some less prominent processes unconnected with blood digestion.

Since the composition of the S2 pocket is the key determinant of substrate specificity of papain-like cysteine peptidases [56], the amino acid sequences of *EnCLs* were aligned (Additional files 4, 5 and 6) and their S2 residues (positions 67, 133, 157, 205) compared. The comparison of various *EnCLs* revealed the existence of three groups differing in the polarity and charge of the S2 pocket. In *EnCL2*, the S2 pocket is strongly hydrophobic and carries a positive charge. *EnCL1*, *EnCL3*, *EnCL4* and *EnCL5* have a moderately hydrophobic S2 pocket with a neutral charge. The least hydrophobic S2 pocket is found in the closely related *EnCL6* orthologs; it has a neutral charge in *EnCL6a* and 6b, and a negative charge in *EnCL6e* (for *EnCL6c* and 6d the complete data are not available). The presence of a negatively charged Asp205 in the S2 pocket may endow *EnCL6e* with the ability to accommodate a positively charged Arg at P2 position of the substrate, which would give this cathepsin L ortholog a cathepsin B-like substrate specificity. Additionally, the group of *EnCL6* orthologs is atypical among cathepsins L by having an amphipathic Gly133 in the S2 pocket. This feature is, however, typical of cathepsins S, and Gly133 can also be found in a few other, yet unclassified, clan CA family C1 peptidases, such as the plerocercoid growth factor of the larval stage of the tapeworm *Spirometra* (Genbank: BAB62718.1) (see the alignment in Additional file 6). Nonetheless, specificity of the S2 pocket in papain-like peptidases is largely determined by the residue 205. Cathepsins S contain at this position a large aromatic non-polar Phe, which implies a short and more restricted pocket. Cathepsins L, on the other hand, usually include at this position branched aliphatic residues, which enables the accommodation of larger aromatic residues [57]. This was not observed in *EnCL6a*, which contains a polar Ser205. It thus seems that diversity in the composition of S2 subsites of *E*. *nipponicum* cathepsins L gives the parasite a wide array of efficient proteolytic tools with diverse affinities to potential P2 positions of protein substrates.

Phylogenetic analysis seems to support a supposition, inferred from the structural data and relative rates of expression, that in *E*. *nipponicum*, cathepsins L may be involved in various biological processes. The two functionally characterised enzymes, *EnCL1* and *EnCL3*, which seem to play a central role in blood digestion by the parasite, form a monophyletic group with *EnCL5*. The other monophyletic clade formed by *EnCL2*, *EnCL4* and *EnCL6* is relatively distant from the first group, thus suggesting involvement in other processes or, in the case of the least proportionally represented transcripts such as *EnCL2* and *EnCL6a*, a more substantial expression of these orthologs in other life stages. The presence of myxosporean-like cathepsins L within the otherwise purely neodermatan clade may lead to a speculation about a lateral gene transfer, since myxosporeans share their fish hosts with monogeneans. Investigation of this possibility would, however, require a thorough analysis that is beyond the scope of the present study. Moreover, it should also be noted that the evolution of genes need not follow the branching structure of species-level evolutionary trees due to mechanisms such as gene duplication and independent gene loss. Particularly, genes of parasites are prone to phylogenetic artefacts caused by attraction between long branches, an effect we tried to mitigate by using site-heterogeneous mixture models [58].

Surprisingly, only one transcript coding for cathepsin B was found in the transcriptome of adult *E. nipponicum.* Amino acid sequence of *EnCB* had shown that this enzyme is a typical representative of cathepsins B, including its active site, composition of the S2 subsite, and the occluding loop responsible for exopeptidase activity. It also contains a ‘haemoglobinase motif’ assigned to helminth cathepsins B with substrate specificity for haemoglobin [47]. In the case of *EnCB*, this motif is slightly modified in its C-terminal region, where the original sequence described for helminth haemoglobinases ‘DWGE’ is replaced by ‘EWGD’ (Additional file 8). Nevertheless, as we experimentally verified, these purely conservative amino acid substitutions did not affect the ability of *EnCB* to cleave haemoglobin. Similar substitutions in the motif were described for schistosome cathepsins B2 (*S. mansoni* and *Trichobilharzia regenti*) that were found only outside the parasites’ digestive system; it is assumed that their functions do not include the digestion of haemoglobin [59, 60]. In contrast to *EnCB*, *T*. *regenti* CB2 degraded haemoglobin at a negligible rate.

In the process of heterologous functional expression in *P. pastoris*, *YrEnCL1* had undergone auto-activation in the cultivation medium by cleaving-off a significant proportion of its pro-sequence and migrated on SDS-PAGE as a 28 kDa band (the value thus differed from the expected ~35 kDa and ~24 kDa of the zymogen and fully processed enzyme, respectively). Other, less abundant bands also occasionally appeared in the gel; mass spectrometry had confirmed they were fragments of *yrEnCL1*, generated probably by auto-degradation (data not shown). This is consistent with the very limited stability of the partially processed enzyme observed during purification. Monospecific *anti-yrEnCL1* antibodies did, however, react with a ~24 kDa band on blots of soluble worm extracts, indicating a full processing of *EnCL1* under natural conditions. The *yrEnCL3*, on the other hand, was obtained as a relatively stable zymogen. It was capable of autocatalytic activation under acidic conditions, but the size of the resulting product, ~28 kDa, was also somewhat higher than the predicted mass of ~24.1 kDa of the mature enzyme. Nevertheless, Edman degradation confirmed an amino acid sequence at the N-terminus of the processed *yrENCL3* which had been predicted by bioinformatical methods, thus indicating that the processing was complete. Monospecific *anti-yrEnCL3* antibodies reacted similarly with a ~28 kDa band on blots of soluble worm extracts. Given that the *EnCL3* does not possess any N-glycosylation motifs, these differences cannot be explained by either natural glycosylation of the enzyme or by N-hyperglycosylation from *P*. *pastoris* in the case of the recombinant enzyme. It could be speculated that the observed shift in Mw of the enzyme was caused by disruption of disulphide bonds stabilizing cathepsin L structure, which resulted in mobility change during electrophoresis [60, 61]. Although *EnCL3* possesses one potential O-glycosylation site in the mature part, the prediction software on the NetOglyc server produces predictions of mucin type GalNAc O-glycosylation sites in mammalian proteins, and the situation may be different in invertebrates. Moreover, we are not aware of any descriptions in literature of a cathepsin L with experimentally confirmed O-glycosylation.

S2 subsite specificity of *yrEnCL1* and *yrEnCL3* was tested over a range of pH with a set of oligopeptide synthetic substrates, varying in their Phe, Leu, Arg, and Pro at P2 position. As predicted from the composition of the S2 pockets, the enzymes were not able to cleave substrates containing Pro or Arg in this position. Consistently with most papain-like peptidases, both enzymes preferred at this position the bulky hydrophobic aromatic Phe and the branched aliphatic Leu, in order Phe > Leu [56]. The enzymes did, however, differ in their pH profiles of activity towards substrates, so that whereas *yrEnCL1* was active only in a narrow pH range with a pH optimum at 5.5, *yrEnCL3* had a broader pH activity profile with an optimum at pH 6. The slightly acidic pH optima correspond to those of cathepsin L-like peptidases of other blood-feeding parasites [61–64].

As expected, activity of both of the recombinant *EnCLs* was inhibited by the general papain-like cysteine peptidase inhibitor E-64. Surprisingly, however, the iCL, a highly potent amphiphilic reversible peptide inhibitor of human cathepsin L [65], had but a little effect on the enzymes’ activity, although it has Leu at the P2 position. One can thus hypothesise that a lower affinity to Trp at P1 position, found also in, e.g. cathepsin L of *Leishmania mexicana* [56], might account for this result. Remarkably, CA-074 (a specific inhibitor of cathepsin B) also partially reduced the activity of both cathepsins L. It has been reported elsewhere [66, 67], however, that CA-074 can decrease the activity of cathepsins L under reducing conditions and that could also explains our results.

Activity profiles, pH optimum, specificity to peptide substrates, inhibition by CA-074 and exopeptidase activity clearly show that *EnCB* is a typical member of cathepsins B group. The pro-enzyme had undergone auto-activation either in the yeast medium or during purification, and enzymatic deglycosylation demonstrated hyperglycosylation of the recombinant enzyme by yeast, which corresponds to the presence of an N-glycosylation motif in the *EnCB* molecule. *Anti-yrEnCB* monospecific antibodies detected native *EnCB* on blots of soluble worm extracts at ~29 kDa, which is adequate to the expected size of the processed enzyme which was probably slightly glycosylated naturally.

Both recombinant *EnCL1* and *EnCL3* efficiently degraded various blood proteins and type I collagen of the connective tissue. Unlike synthetic peptide substrates, macromolecular substrates (except for fibrinogen) were cleaved only at pH 4.5 by *yrEnCL3*, while *yrEnCL1* hydrolysed all macromolecular substrates at pH 4.5–6.0. Activity at low pH values is in accordance with the site of expression of both genes encoding the enzymes that was localised by *in situ* hybridisation in the haematin (digestive) cells, and with the presumed intracellular digestion of haemoglobin in the endolysosomal vesicles where pH below 4.5 would be expected [68]. Both enzymes, however, were also immunolocalised in the lumen of the parasite’s gut. For other blood-feeding helminths, it has been estimated that the pH in the gut lumen is only slightly acidic (*c.*5.5–6.5) [43, 69, 70], and digestion therefore takes place in a local microenvironment between the lamellae of the digestive cells where the pH is more acidic (~4.5), as documented for *F. hepatica* [71]. Haematin cells of another studied blood-feeding diplozoid monogenean, *Paradiplozoon homoion*, also contain numerous lamellae on the side facing the lumen of the gut. Host erythrocytes are lysed in proximity of the haematin cells and released haemoglobin is taken in by lamellae of the cells via pinocytosis [9]. These facts, together with presence of *EnCL1* and *EnCL3* in excretory/secretory products of the worms [12], and with the results of immunolocalisation, imply that in diplozoid monogeneans extracellular, luminal digestion of blood can take place. Moreover, if the enzymes are released from the gut lumen into host circulation, they could potentially participate in some host-parasite interactions by affecting, e.g. fibrinolysis (they cleaved fibrinogen in vitro) or immune response (degradation of immunoglobulins).

Surprisingly, both *EnCLs* hydrolysed collagen type I. This ability was attributed in human body primarily to family C1 lysosomal cysteine peptidase cathepsin K. Because of acceptance of Pro in the P2 position, cathepsin K can completely degrade collagen by cleaving within the repeated Gly-Pro-Xaa motifs that occur in the triple helix of collagen [54]. The ability to accommodate substrates with P2 proline has also been observed in some cathepsins L of *F. hepatica*, namely *FhCL2*, *FhCL3* and *FhCL5* [50, 55, 72]. On the other hand, neither *yrEnCL1* nor *yrEnCL3* cleaved synthetic substrates GPR and PR. So, though both enzymes degraded native collagen, they probably do not possess intrahelical activity and this likely makes them less effective collagenases. Such a phenomenon has been described in human cathepsin L and *FhCL1* [51, 54].

During the localisation of expression of *EnCL1* and *EnCL3* by RNA *in situ* hybridisation, sense-RNA probes used as controls had shown similar reaction as antisense probes. It is known from the literature that over 30% of genes transcribed in humans also have antisense transcription [73], and antisense transcripts have been described for genes in a diverse group of eukaryotes [74–76]. The use of a specific strand RT-PCR has revealed that the *EnCL1* and *EnCL3* encoding genes also have naturally present antisense transcripts. Since the cathepsin L-like peptidases play important roles during the life of the monogeneans, antisense transcripts may be associated with the regulation of gene transcription [77].

It has originally been thought that *EnCB* is also involved in the proteolytic cascade of blood digestion in *E*. *nipponicum* [12]. In haematophagous parasites, many cathepsins B, together with other peptidases of the digestive cascades, function as important enzymes with a mixed mode of action, as endopeptidases that cleave proteins into smaller fragments and as exopeptidases (peptidyldipeptidases, carboxypeptidases) that remove dipeptides from the C-termini of those protein fragments due to the presence of the occluding loop [78]. Immunofluorescence with monospecific *anti-yrEnCB* antibodies clearly showed the localisation of *EnCB* in vesicular structures in vitelline cells, but its presence in the digestive tract has not been detected at all. We could thus speculate on its function in vitellogenesis. The presence of cysteine peptidases in the reproductive system has been detected also in other helminths [79–82], but their role in worms’ reproduction is still speculative. The lack of exopeptidase activity in the gut due to the absence of cathepsin B may be compensated by the activity of cathepsins C; at least three different transcripts of these dipeptidyl peptidases (aminopeptidases) were found in *E*. *nipponicum* transcriptome (not shown).

## Conclusions

To our knowledge, this work represents the first comprehensive exploration and functional annotation of a group of peptidases from a monogenean transcriptome. It also describes the very first functional characterisation of recombinant cysteine peptidases of monogenean origin. The lack of experimental data from other monogeneans, particularly those of a high economic importance (such as *Gyrodactylus salaris*, *Benedenia* spp.), unfortunately precludes the possibility of drawing any comparisons. We have shown that the haematophagous monogenean *E*. *nipponicum* possesses a wide range of endopeptidases of cathepsin L type, most of which are highly expressed in adult worms. Some of these enzymes possess structural features rather unusual in cathepsins L. It holds true especially for the composition of S2 subsite of the active site, which is among the studied enzymes rather variable and includes even substitutions typical more of cathepsins B and cathepsins S. This expands our understanding of the structural diversity of cathepsins L in general. In contrast to the rich world of cathepsins L, the presence of just one cathepsin B in the worms was somewhat surprising. Despite its ability to hydrolyse haemoglobin in vitro, *EnCB*, due to its localisation in the vitelline cells of the parasite, does not seem to be involved in digestion. On the other hand, functional characterisation and localisation has revealed that *EnCL1* and *EnCL3* are important in the digestion of blood. Despite previous claims, it seems that the digestive process takes place not only within the intracellular lysosomal cycle, but at least in part also outside the gastrodermis, in the lumen of the gut. From there, the *EnCLs* may be eventually released into the blood circulation of the host and participate in elicitation of immune response and/or immunomodulation. Genomic data, currently available only for the mucophagous polyonchoinean *G. salaris* and the haematophagous heteronchoinean *Protopolystoma xenopodis*, and transcriptomic data from the mucophagous polyonchoinean *Neobenedenia melleni*, open a possibility for future comparative biochemical research of the two trophic strategies characteristic of the monogeneans. For example, preliminary data explorations show that mucus feeders may employ elastase-like serine peptidases that have not been found in blood-feeders (Mikeš, unpublished). Further research is likely to provide data interesting from an evolutionary point of view and information essential to understanding parasite-host interactions on a molecular level in this group of parasites, which would be of interest especially in context of pathogenesis and fish immunity. Although parasite peptidases have often been seen as promising targets for the development of vaccines or new inhibitor-based remedies, in the case of monogeneans we are somewhat sceptical regarding this possibility. The main reasons why we do not believe this is a promising direction are the likely costs linked to a complicated handling of large numbers of individual fish hosts in aquaculture, difficulties with administering a correct dosage of medication (peptidase inhibitors) in an aquatic environment (assuming that a peroral formula would even be available) and the necessity of repeated administering in order to reach a lethal effect on the parasites. At the moment, most methods applied against monogeneans rely on mass treatment of farmed fish by readily available, low-cost chemical biocides, such as hydrogen peroxide, or antihelminthics, such as praziquantel [83].

## Additional files

Additional file 1: Primers for the expression of *yrEnCL1*, *yrEnCL3*, and *yrEnCB* in *P. pastoris. (PDF 239 kb)*

Additional file 2: Primers for the expression of *brEnCL1* in *E. coli*. (PDF 83 kb)

Additional file 3: Specific *EnCL1/EnCL3* primers for the synthesis of probes for RNA *in situ* hybridisation. (PDF 84 kb)

Additional file 4: Amino acid sequence alignment of *E*. *nipponicum* cathepsins L with cathepsins L of *F. hepatica*. Signal peptides and pro-sequences were omitted. Numbers at the end of the lines show amino acid numbering of particular mature parts of the enzymes. Catalytic triad of the active site (C, H, N) is marked by triangles. Conserved motifs around active site residues are shaded in grey. Residues within the S2 subsite of the active site involved in determining the substrate specificity are shaded in black and indicated with numbers (papain numbering). Tripeptides of potential N-glycosylation sites are boxed. Predicted O-glycosylated residues are marked by grey squares. The alignment was made using the sequences of *F. hepatica* mature cathepsins L1 (GenBank: AAT76664.1) and L3 (GenBank: CAC12807.1). (PDF 1949 kb)

Additional file 5: Multiple alignment of all the complete/incomplete amino acid sequences of *E*. *nipponicum* cathepsins L inferred from the transcriptome of adult worms. Signal peptides are shaded in dark grey, position of the pro-region cleavage site is marked by arrows. ERFNIN- and GNFD-like motifs are underlined and indicated by underlined headings. The catalytic triad of the active site (C, H, N) is marked by triangles. Conserved motifs around active site residues are shaded in light grey. Residues within the S2 subsite of the active site involved in determining the substrate specificity are shaded in black and indicated with numbers (papain numbering). Tripeptides of potential N-glycosylation sites are boxed. Predicted O-glycosylated residues are marked by grey squares. (PDF 5783 kb)

Additional file 6: Composition of S2 subsites of the active sites shown in the alignment of parts of amino acid sequences of *E*. *nipponicum* cathepsin L orthologs 6a, 6b and 6e with human cathepsin S and with plerocercoid growth factor of *Spirometra* tapeworm. The catalytic triad of the active site (C, H, N) is marked by triangles. Conserved motifs around active site residues are shaded in grey. Residues within the S2 subsite of the active site involved in determining the substrate specificity are shaded in black and indicated with numbers (papain numbering). *Abbreviations*: PGF, *Spirometra erinaceieuropaei* plerocercoid growth factor (Genbank: BAB62718.1); *HsCS*, human cathepsin S (Genbank: AAC37592). (PDF 1406 kb)

Additional file 7: Phylogram showing relationships between *E. nipponicum* cathepsins L and cathepsins L of other selected organisms. Unrooted maximum-likelihood tree of selected *E. nipponicum* cathepsins L inferred using the best-fit model (LG + C60 + G). Ultrafast bootstrap supports and posterior probabilities are shown. The leaf descriptions contain the sequence ID, genus and taxonomic placement. (PDF 4260 kb)

Additional file 8: Amino acid sequence alignment of *E*. *nipponicum* cathepsin B with cathepsins B of *S. mansoni*. Whole zymogens including a signal peptide were aligned. Numbers at the end of the lines show amino acid numbering of the mature parts of particular enzymes. Position of the pro-region cleavage site is marked by an arrow. The catalytic triad of the active site (C, H, N) is marked by triangles. Conserved motifs around active site residues are shaded in grey. Tripeptides of potential N-glycosylation sites are boxed. Predicted O-glycosylated residues are marked by grey squares. The occluding loop typical of cathepsins B is underlined. The ‘haemoglobinase motif’ ascribed to cathepsins B with an assumed function in haemoglobinolysis in blood-feeding helminths, is shaded in black (it is modified in *SmCB2* and *EnCB*). Residues within the S2 subsite of the active site involved in determining the substrate specificity are marked by a black dot and indicated with number (papain numbering). The alignment was made using sequences of *Schistosoma mansoni* cathepsins B1 (GenBank: CAD44624.1) and B2 (GenBank: XP_018651608.1). (PDF 1923 kb)

## Abbreviations

AMC: 7-amino-4-methylcoumarin
BSA: Bovine serum albumin
Bz-Gly-His-Leu: Benzoyl-glycinyl-histidinyl-leucine
CA-074: N-[L-3-trans-propylcarbamoyloxirane-2-carbonyl]-Ile-Pro-OH
CBB: Coomassie Brilliant Blue
CPB: Citrate/phosphate buffer
DCG-04: fluorescent labelled probe, derivate of inhibitor E-64
DTT: Dithiothreitol
E-64: (L-trans-epoxysuccinyl–leucylamido [4-guanidino] butane)
*EnCB*: *Eudiplozoon nipponicum* cathepsin B
*EnCL*: *Eudiplozoon nipponicum* cathepsin L
ESP: Excretory secretory products
FPLC: Fast protein liquid chromatography
FR: Substrate for serine and cysteine peptidases Z-Phe-Arg-AMC
GPR: Substrate for serine peptidases Z-Gly-Pro-Arg-AMC
His-tag: polyhistidine-tag (6xHis)
ICL: Reversible inhibitor of cathepsin L(Arg-Lys-Leu-Leu-Trp-NH2)
LR: Substrate for serine and cysteine peptidases Z-Leu-Arg-AMC
MES: (2-N-morpholino) ethanesulfonic acid
Ni-NTA: Nickel-nitrilotriacetic acid
PBS- TX100: Phosphate buffered saline containing 0.3% Triton X100
PBS: Phosphate buffered saline
PBS-T: Phosphate-buffered saline containing 0.05% Tween 20
PR: Substrate for serine peptidases Z-Pro-Arg-AMC
PVDF: Polyvinylidene difluoride
RACE-PCR: Rapid amplification of cDNA ends
RR: Substrate for cathepsin B Z-Arg-Arg-AMC
RT: Room temperature
RT-PCR: Reverse transcription-PCR
SDS-PAGE: Sodium dodecyl sulfate polyacrylamide gel electrophoresis
SSC: Saline-sodium citrate buffer
