# Supplementary material for: Cysteine peptidases of Eudiplozoon nipponicum: a broad repertoire of structurally assorted cathepsins L in contrast to the scarcity of cathepsins B in an invasive species of haematophagous monogenean of common carp
Source: Parasit Vectors. 2018 Mar 6;11:142. doi: 10.1186/s13071-018-2666-2 (PMC5840727; doi:10.1186/s13071-018-2666-2)
Supplement: Supplementary file 1 — Primers for the expression of yrEnCL1, yrEnCL3, and yrEnCB in P. pastoris. (PDF 239 kb) [file 13071_2018_2666_MOESM1_ESM.pdf]

yrEnCL3Fwd	ATACTCGAG**AAAAGA**GCTGATCAAAATGAATCACCCTTCCAGA
yrEnCL3Rev	ATGCGGCCGC**TTA**ATGATGATGATGATGATGAACGAGAGGATATGAGG
yrEnCL1Fwd	ATACTCGAG**AAAAGA**ATGGCAGGTCAGGATCACTGGGCTTCATTCAAG
yrEnCL1Rev	ATGCGGCCGC**CTA***ATGATGATGATGATGATG*TACGAGTGGATAGCTTGC
yrEnCBFwd	ATACTCGAG**AAAAGA**CGAGTTTTCCACAGTCAG
yrEnCBRev	AATGCGGCCGCTTAC*ATGATGATGATGATGATG*ATAAGTTTCGGTATCCC
