# Supplementary material for: Cysteine peptidases of Eudiplozoon nipponicum: a broad repertoire of structurally assorted cathepsins L in contrast to the scarcity of cathepsins B in an invasive species of haematophagous monogenean of common carp
Source: Parasit Vectors. 2018 Mar 6;11:142. doi: 10.1186/s13071-018-2666-2 (PMC5840727; doi:10.1186/s13071-018-2666-2)
Supplement: Supplementary file 2 — Primers for the expression of brEnCL1 in E. coli. (PDF 83 kb) [file 13071_2018_2666_MOESM2_ESM.pdf]

brCL1Fwd	CATAGAGCTCATGGCAGGTCAGGATCACTGGGCT
brCL1Rev	GATACTCGAGTACGAGTGGATAGCTTGCGCATGTTGCCAC
