# Supplementary material for: Cysteine peptidases of Eudiplozoon nipponicum: a broad repertoire of structurally assorted cathepsins L in contrast to the scarcity of cathepsins B in an invasive species of haematophagous monogenean of common carp
Source: Parasit Vectors. 2018 Mar 6;11:142. doi: 10.1186/s13071-018-2666-2 (PMC5840727; doi:10.1186/s13071-018-2666-2)
Supplement: Supplementary file 3 — Specific EnCL1/EnCL3 primers for the synthesis of probes for RNA in situ hybridisation. (PDF 84 kb) [file 13071_2018_2666_MOESM3_ESM.pdf]

EnCL1Fwd	GAC ACA ACA CGA AGA ACA ATT TTT GAG
EnCL1Rev	AGT GCT GAC CTT CCA ATG AC
EnCL3Fwd	TTT GTA TCA TCG GCT GTT TCA GTG
EnCL3Rev	CTA CAT CTG TAG GCA ACT TGA AAT CT
