# Supplementary material for: Cysteine peptidases of Eudiplozoon nipponicum: a broad repertoire of structurally assorted cathepsins L in contrast to the scarcity of cathepsins B in an invasive species of haematophagous monogenean of common carp
Source: Parasit Vectors. 2018 Mar 6;11:142. doi: 10.1186/s13071-018-2666-2 (PMC5840727; doi:10.1186/s13071-018-2666-2)
Supplement: Supplementary file 4 — Amino acid sequence alignment of E. nipponicum cathepsins L with cathepsins L of F. hepatica. Signal peptides and pro-sequences were omitted. Numbers at the end of the lines show amino acid numbering of particular mature parts of the enzymes. Catalytic triad of the active site (C, H, N) is marked by triangles. Conserved motifs around active site residues are shaded in grey. Residues within the S2 subsite of the active site involved in determining the substrate specificity are shaded in black and indicated with numbers (papain numbering). Tripeptides of potential N-glycosylation sites are boxed. Predicted O-glycosylated residues are marked by grey squares. The alignment was made using the sequences of F. hepatica mature cathepsins L1 (GenBank: AAT76664.1) and L3 (GenBank: CAC12807.1). (PDF 1949 kb) [file 13071_2018_2666_MOESM4_ESM.pdf]

```
                                        ▽
EnCL1       LPERVDWRDKGLVTPVKNQGQCGSCWAFSTTGSLEGQHFRKTGKLLSLSEQQLVDCSSAF 60
EnCL2       LPTNVDWRHSGFVNPPKRQGECRSSYAFSTVASIETYWAKKSGQLLQLSEQQLMDCATSQ 60
EnCL3       LPTDVDWRNDGAVTDVKNQGQCGSCWAFSTTGSLEGQHFRKTGNLVSLSEQQLVDCSSSY 60
EnCL4       LPKSVDWRTFGVINNVKNQGRCGSCYAFATVCTIESHYAIKTSQLINLSEQQIVDCAIDE 60
EnCL5       LPDHVDWRTKGAVTDVKDQGQCGSCWAFSTTGSLEGQHFRKTGKLVSLSEQQLTDCSSSY 60
EnCL6a      LPQSIDWRNFGVVNRVKDEGNCGSCYAFATVCTIESHYAIKTGQLLKLSEQQIVDCAGEE 60
EnCL6e      LPQSVDWRNFGVINRIKDQGDCGSCYAFATVCTIESHYAIKTSHLLRLSEQQIVDCAIDE 60
FhCL1       VPDKIDWRESGYVTGVKDQGNCGSCWAFSTTGTTEGQYMKNERTSISFSEQQLVDCSGPW 60
FhCL3       VPASIDWRQYGYVTEVKDQGQCGSCWAFSPVGAIEGQYVKKFQNQTLFSEQQLVDCTRRF 60
            :*  :***  * :.  * :*  * *.:**: . : *     :      :****: **:

                    67
EnCL1       GNHGCNGGLFDFAFKYVQDSGGITTEDLYPYVSGVIQKAHDVCSYNPDMCKATCTGWVDI 120
EnCL2       KMDPCKDNWPNYAFDYIILN-GLTTEDNYSYRA-----KKGQCKEIQIKDVVWIYNYVDL 114
EnCL3       GNMGCNGGLMDNAFAYIKATNGIDYEDKYPYVSGDTGSAEDTCYFKEEDIGAVDTGYVDI 120
EnCL4       GDEGCNGGLMDFSYDYVLIR-GLTKAQNYPYKA-----KSGVCKDDKIEPAVHIHSYVDL 114
EnCL5       GNQGCNGGLVDQAFQYVRDCGGLQSENSYPYISGATSQA-ASCNFDPNKIAATCTGFVDL 119
EnCL6a      GDEACDGGLPDFTYDYVFFR-GLTTEKNYPYKE-----KMGICMDHKYKPAVHIHNYVDL 114
EnCL6e      GDDACDGGLPDYSYDYVLIR-GLTTEKDYPYKE-----GMTICKDDKFKPAVHIHSYVDL 114
FhCL1       GNNGCGGGLMENAYEYLKQF-GLETESSYPYTA-----VEGQCRHSKQLGVAKVTGYYTV 114
FhCL3       GNHGCGGGWMENAYKYLKNS-GLETASDYPYQG-----WEYQCQYRKELGVAKVTGAYTV 114
                * ..  : :: *:    *:   . * *           *            .   :

                    133                                   157 ▽
EnCL1       PSKDSKALMYAVATIGPISIAINAMGPGFMQYKSGIYNPPSCPGDFSDLDHAVLLVGYGT 180
EnCL2       PANDEHAMQLTLALLGPLSVVLEAA-PDLQFYAGGVYNSVQCHDQEETFDHAMNIVGYGT 173
EnCL3       PTEDEAALQEAVANVGPVSVAINAGRADFMMYKQGIYKPDECPGQMNDLDHGVLVVGYGS 180
EnCL4       PSNDEYALKLSLALLGPVAVAIDAE-NDFRHYKKGVFNSTICDDDPETLDHALTVIGYGT 173
EnCL5       PEGDEAALMHALATTGPVSIAINAEPADFMSYRQGIYENPMARGDRSNLDHAVLCIGYGS 179
EnCL6a      PVEDEYALKLSVALLGPVAVGIDAD-NNFKFYKGGVFNSTMCDDDLATLDHAAVVIGYGN 173
EnCL6e      PAQDEYAMKLSVALMGPVAVGIDAE-NTFRFYKGGVFNSTMCHDDPSALDHAVVVIGYGT 173
FhCL1       HSGSEVELKNLVGAERPAAVAVDVE-SDFMMYRSGIYQSQTCSPL--SVNHAVLAVGYGT 171
FhCL3       HSGDEMKLMPMVRKKGPAAAAVDAQ-PDFYMYESGIFQSQYCSSR--RVTHAVLAVGHGT 171
               ..  :   :    * :  ::.    :  *  *:::   .      . *.   :*:*.

                         ▽                          205
EnCL1       Q--NGLNYWIVKN-SWSEKWGENGYVRICRD-GRNLCGVATCASYPLV----     224
EnCL2       DEQSGQNYWLVRN-SWGESWGENGYIRIARTTPNNLCGIAERPSYPLV----     220
EnCL3       E--NGQDYWIVKN-SWGPDWGESGYIRMARN-SGNLCGIATAASYPLV----     224
EnCL4       DRVTGQDYWLVRN-SWGSSWGENGYIRIARTNPANLCGITDAASFPLV----     220
EnCL5       E--NGKKYWLVKN-SWSGAWGEQGYIRIARD-DRNICGVTTLASYPLV----     223
EnCL6a      DKITGQDYWLVRN-SWGASWGESGYVRYARTNPHNLCGITDSASFPLV----     220
EnCL6e      DEVSGQDYWLVRNNSCGTSWGESGYIRYARTNPQNLCGITDDASFPLV----     221
FhCL1       Q--GGTDYWIVKN-SWGLSWGERGYIRMVRN-RGNMCGIASLASLPMVARFP     219
FhCL3       E--SGTDYWILKN-SWGKWWGEDGYMRFARN-RGNMCAIASVASVPMVERFP     219
            :   * .**:::* * .  *** **:*  *    *:*.::   * *:*
```
