# Supplementary material for: Cysteine peptidases of Eudiplozoon nipponicum: a broad repertoire of structurally assorted cathepsins L in contrast to the scarcity of cathepsins B in an invasive species of haematophagous monogenean of common carp
Source: Parasit Vectors. 2018 Mar 6;11:142. doi: 10.1186/s13071-018-2666-2 (PMC5840727; doi:10.1186/s13071-018-2666-2)
Supplement: Supplementary file 6 — Composition of S2 subsites of the active sites shown in the alignment of parts of amino acid sequences of E. nipponicum cathepsin L orthologs 6a, 6b and 6e with human cathepsin S and with plerocercoid growth factor of Spirometra tapeworm. The catalytic triad of the active site (C, H, N) is marked by triangles. Conserved motifs around active site residues are shaded in grey. Residues within the S2 subsite of the active site involved in determining the substrate specificity are shaded in black and indicated with numbers (papain numbering). Abbreviations: PGF, Spirometra erinaceieuropaei plerocercoid growth factor (Genbank: BAB62718.1); HsCS, human cathepsin S (Genbank: AAC37592). (PDF 1406 kb) [file 13071_2018_2666_MOESM6_ESM.pdf]

```
EnCL6a          LPQSIDWRNFGVVNRVKDEGNCGSCYAFATVCTIESHYAIKTGQLLKLSEQQIVDCAG-E
EnCL6b          -----------------------------------------------SEQQICSTCAI-D
EnCL6e          LPQSVDWRNFGVINRIKDQGDCGSCYAFATVCTIESHYAIKTSHLLRLSEQQIVDCAI-D
PGF             LPDSVNWRERGAVTSVKNQGQCGSCWSFSANGAIEGAIQIKTGALRSLSEQQLMDCSW-D
HsCS            LPDSVDWREKGCVTEVKYQGSCGACWAFSAVGALEAQLKLKTGKLVSLSAQNLVDCSTEK
                                                                . *    *:  .

                        67
EnCL6a          EGDEACDGGLPDFTYDYVF-FRGLTTEKNYPYKEKMGICMDHKYKPAVHIHNYVDLPVED
EnCL6b          EGDDACDGGLPDFSYDYVL-IRGLTTEKDYPYKDGMTICKDDKFKPAVHIHSYVDLPAQD
EnCL6e          EGDDACDGGLPDYSYDYVL-IRGLTTEKDYPYKEGMTICKDDKFKPAVHIHSYVDLPAQD
PGF             YGNQGCNGGLMPQAFQYAQ-RYGVEAEVDYRYTERDGVCRYRQDLVVANVTGYAELPEGD
HsCS            YGNKGCNGGFMTTAFQYIIDNKGIDSDASYPYKAMDLKCQYDSKYRAATCSKYTELPYGR
                 *:..*:**:   :::*     *: ::  .* *.     *   .         *.:**

                               133                        157
EnCL6a          EYALKLSVALLGPVAVGIDAD-NNFKFYKGGVFNSTMCDDDLATLDHAAVVIGYGNDKIT
EnCL6b          EYAMKLSVALMGPVAVGIDAE-NTFRFYKGGVFNSTMCDDDPSTLDHAVVVIGYGTDEVS
EnCL6e          EYAMKLSVALMGPVAVGIDAE-NTFRFYKGGVFNSTMCHDDPSALDHAVVVIGYGTDEVS
PGF             EGGLQRAVATIGPISVGIDAADPGFMSYSHGVFVSKTCS--PYAIDHGVLVVGYGA--EN
HsCS            EDVLKEAVANKGPVSVGVDARHPSFFLYRSGVYYEPSCT--Q-NVNHGVLVVGYGD--LN
                *  :: :**  **::**:**    *  *  **: .  *      ::*..:*:***    .

                                                    205
EnCL6a          GQDYWLVRN-SWGASWGESGYVRYARTNPHNLCGITDSASFPLV
EnCL6b          GQDYWLVRN-SWSASWGESGYMRYARTNPHNLCGITDMASFPLV
EnCL6e          GQDYWLVRNNSCGTSWGESGYIRYARTNPQNLCGITDDASFPLV
PGF             GEAYWLVKN-SWGSSWGEGGYVKMAR-NRNNMCGIASMASYPTV
HsCS            GKEYWLVKN-SWGHNFGEEGYIRMAR-NKGNHCGIASFPSYPEI
                *: ****:* * . .:** **:: ** *  * ***:.  *:* :
```
