# Supplementary figures and images for: Cysteine peptidases of Eudiplozoon nipponicum: a broad repertoire of structurally assorted cathepsins L in contrast to the scarcity of cathepsins B in an invasive species of haematophagous monogenean of common carp
Source: Parasit Vectors. 2018 Mar 6;11:142. doi: 10.1186/s13071-018-2666-2 (PMC5840727; doi:10.1186/s13071-018-2666-2)

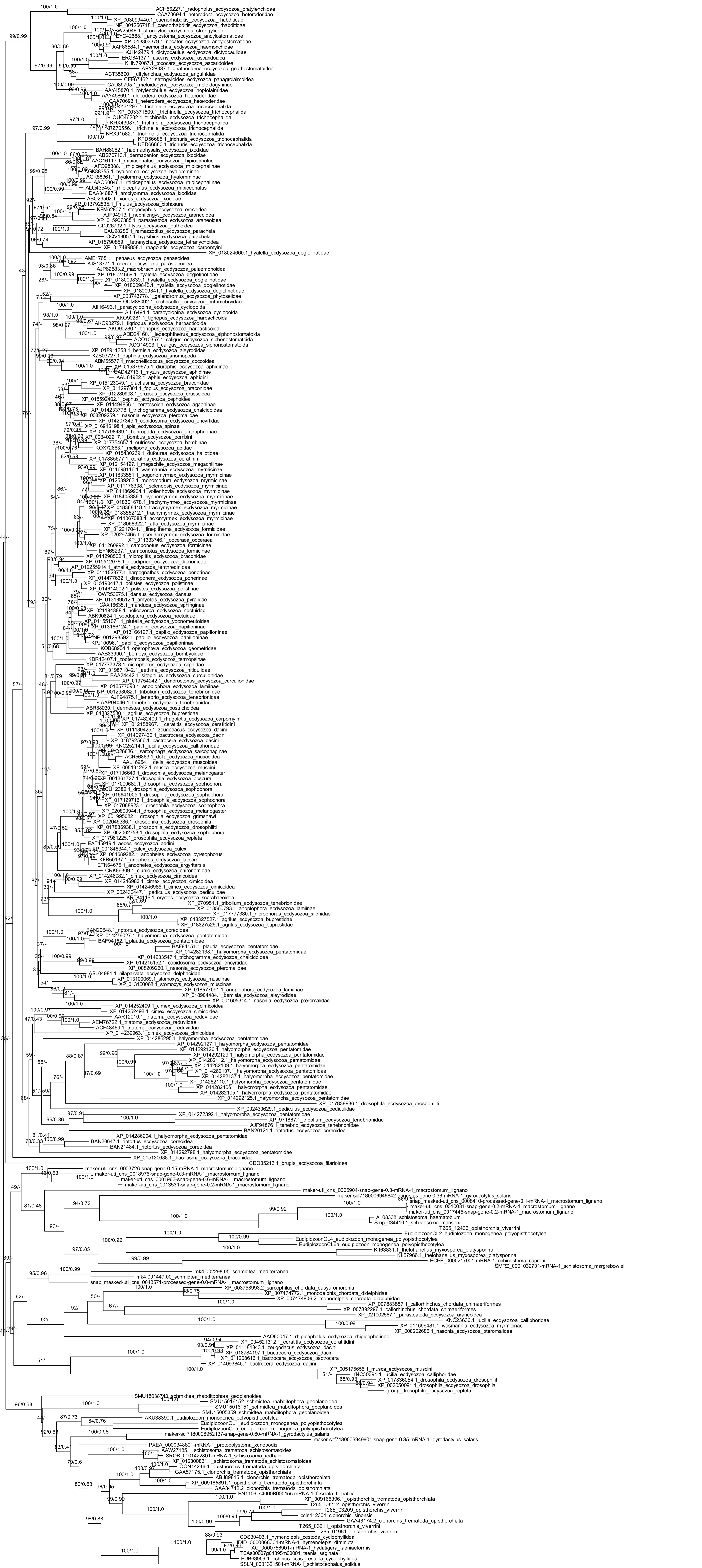

Supplement: Supplementary file 7 — Phylogram showing relationships between E. nipponicum cathepsins L and cathepsins L of other selected organisms. Unrooted maximum-likelihood tree of selected E. nipponicum cathepsins L inferred using the best-fit model (LG + C60 + G). Ultrafast bootstrap supports and posterior probabilities are shown. The leaf descriptions contain the sequence ID, genus and taxonomic placement. (PDF 4260 kb) [file 13071_2018_2666_MOESM7_ESM.pdf]
