# Supplementary material for: Cysteine peptidases of Eudiplozoon nipponicum: a broad repertoire of structurally assorted cathepsins L in contrast to the scarcity of cathepsins B in an invasive species of haematophagous monogenean of common carp
Source: Parasit Vectors. 2018 Mar 6;11:142. doi: 10.1186/s13071-018-2666-2 (PMC5840727; doi:10.1186/s13071-018-2666-2)
Supplement: Supplementary file 8 — Amino acid sequence alignment of E. nipponicum cathepsin B with cathepsins B of S. mansoni. Whole zymogens including a signal peptide were aligned. Numbers at the end of the lines show amino acid numbering of the mature parts of particular enzymes. Position of the pro-region cleavage site is marked by an arrow. The catalytic triad of the active site (C, H, N) is marked by triangles. Conserved motifs around active site residues are shaded in grey. Tripeptides of potential N-glycosylation sites are boxed. Predicted O-glycosylated residues are marked by grey squares. The occluding loop typical of cathepsins B is underlined. The ‘haemoglobinase motif’ ascribed to cathepsins B with an assumed function in haemoglobinolysis in blood-feeding helminths, is shaded in black (it is modified in SmCB2 and EnCB). Residues within the S2 subsite of the active site involved in determining the substrate specificity are marked by a black dot and indicated with number (papain numbering). The alignment was made using sequences of Schistosoma mansoni cathepsins B1 (GenBank: CAD44624.1) and B2 (GenBank: XP_018651608.1). (PDF 1923 kb) [file 13071_2018_2666_MOESM8_ESM.pdf]

```
SmCB1  ------------------------------------MLTSI----LCIASLITFLEAHI
SmCB2  MN-----QYSCYLLQLY-----------------------IIILLSYG-----TLNEIDA
EnCB   MRLHSHCSVICVVLVLLAVGLGVQARVFHSQPGTGITRLRQLLREGFSNVKRDNYIKPEG
                                                :              .  : .

SmCB1  SVKNEKFEPLSDDIISYINEHPNAGWRAEKSNRFHSLDDARIQMGARREEPDLRRTRRP-
SmCB2  RRHKRMYQPLSMELINFINYEANTTWKAAPTTRFRTVSDIRRMLGALPDPNGEQLETLCT
EnCB   KLYKPIFTPLSENIINAVN-NANTTWKAGPTTRFNSISALRSQLGVVPDPNGRRLPTKCS
          :  : *** ::*. :* . *: *:*  :.**.::.  *  :*.  :  .  :

SmCB1  -TVDHNDWNVEIPSSFDSRKKWPRCKSIATIRDQSRCGSCWAFGAVEAMSDRSCIQSGGK  49
SmCB2  --GYIS---DELPKSFDARVEWPHCPSISEIRDQSSCGSCWAFGAVEAMSDRICIKSKGK  49
EnCB   TRGYLNEEYQNLPETFDARKAWPNCPTISQIRDQSTCGSCWAFGAVESMSDRICIHSRGN  49
            .     ::*.:**:*  **.* :*: ***** ***********:**** **:* *:

SmCB1  QNVELSAVDLLSCCE-SCGLGCEGGILGPAWDYWVKEGIVTGSSKENHTGCEPYPFPKCE  108
SmCB2  HKPFLSAENLVSCCS-SCGMGCNGGFPHSAWLYWKNQGIVTGDLYNTTNGCQPYEFPPCE  108
EnCB   LKPELSAEDLVSCCGEFCGDGCNGGFPQQAWLYWVRHGIVTGGEYHSTDCCRPYEFPPCD  109
        :  *** :*:***   ** **:**:    ** ** ..*****.   ..   *.** ** *:

SmCB1  HHTKGKYPPCGSKIYKTPRCKQTCQKKYKTPYTQDKHRGKSSYNVKNDEKAIQKEIMKYG  168
SmCB2  HHVIGPLPSCDGDV-ETPSCKTNCQPGYNIPYEKDKWYGEKVYRIHSNPEAIMLELMRNG  167
EnCB   HHVNGTLIPCQGEV-PTPICKHDCQPSYHKSYKADKYYGKESYTVVGE-LHIMRELMENG  167
       **. *   *  ..:  ** **  ** *:  *  **  *:. * : .:  *  *:*. *

SmCB1  PVEAGFTVYEDFLNYKSGIYKHITGETLGGHAIRIIGWGVENKTPYWLIANSWNEDWGEN  228
SmCB2  PVEVDFEVYADFPNYKSGVYQHVSGALLGGHAVRLLGWGEENNVPYWLIANSWNSDWGDK  227
EnCB   PLEVDFEVYADFPNYKSGVYQHVAGALLGGHAVRLLGWGTENGVKYWLIANSWNTEWGDK  227
       *:*..* ** ** *****:*:*:*  *****:*::*** **  . ********* :**::

                         205
SmCB1  GYFRIVRGRDECSIESEVTAGRIN---  252
SmCB2  GYFKIVRGKNECGIESDVNAGIPKIKN  254
EnCB   GLFKIRRGTNECGIESDVVGGIPKL--  252
       * *:* ** :**.***:* .*  :
```
