# Supplementary material for: Cysteine peptidases of Eudiplozoon nipponicum: a broad repertoire of structurally assorted cathepsins L in contrast to the scarcity of cathepsins B in an invasive species of haematophagous monogenean of common carp
Source: Parasit Vectors. 2018 Mar 6;11:142. doi: 10.1186/s13071-018-2666-2 (PMC5840727; doi:10.1186/s13071-018-2666-2)
Supplement: Supplementary file 5 — Multiple alignment of all the complete/incomplete amino acid sequences of E. nipponicum cathepsins L inferred from the transcriptome of adult worms. Signal peptides are shaded in dark grey, position of the pro-region cleavage site is marked by arrows. ERFNIN- and GNFD-like motifs are underlined and indicated by underlined headings. The catalytic triad of the active site (C, H, N) is marked by triangles. Conserved motifs around active site residues are shaded in light grey. Residues within the S2 subsite of the active site involved in determining the substrate specificity are shaded in black and indicated with numbers (papain numbering). Tripeptides of potential N-glycosylation sites are boxed. Predicted O-glycosylated residues are marked by grey squares. (PDF 5783 kb) [file 13071_2018_2666_MOESM5_ESM.pdf]

```
CL1     -----------------------------------------------MAGQDHWASFK
CL2     MPELHFSTV----VVF-----------------------VVFSVASGAIQPSLYNEWSQWK
CL3     MGSFAKMFVAASFVLCFCIIGCFSADQNESPFQ--KNFIINSNTASKGVDYVSQSWSMFK
CL4     MKNIEFYSL----TIC------FSAI-----LLSV---CATRISRSNINTTIKSEWKAWK
CL5     ----------MSFCCGCCDKGKFEEEDE-SILRPQTKLLHEHLHQSLDKGDYDKCWSMFK
CL6a    MNTVQLGLL----LIS------VNAN-----LLLG---RSLRISEISADCDIRSEWNAWK
CL6b    ------------------------------------------------------------
CL6c    MNTTQFCLF----LLS------VGAS-----SLLV---SSLRINDINADSSIRSEWNAWK
CL6d    MNTAQTCLF----LIS------IGAS-----FLHV---NSLRINEINADSSIRREWNAWK
CL6e    ------------------------------------------------------------

                   E    R   F N   I   N                GN F D
CL1     SQHAKNYDDEE-DTTRRTIFETNLAAVEHHNALFNTGRADYSLALNHLSDWKDHELLCLR
CL2     AMFNKSYATATIEAEKLSVWMENRKHILAHNQN---QKFFYRLGLTEFADKTKQEFLSQY
CL3     NFFKRNFENAIEEGERFFIFARNFFMISSHNAEYASGKKIYELTLNKFSDAKESELMKLR
CL4     TTYEKSYDTQEEEEHRFATWLDIREKVRQHNAEYEAGKLSYGMELNQFSDESDEEFALRY
CL5     KLHQKSYEESEEESWRFELFKCKVNDVVAHNVRYKQGHVSYELGINHTADMTSAEKNILR
CL6a    LSYGKSYESHE-EEQRFAIWLSNRDRINSHNRKFEAGSLSYSLELNQFGDESDDEFAIRH
CL6b    ------------------------------------------------------------
CL6c    LTYGKSYESHE-EEKRFSIWLDNREKIKRHNRKYEDGSLSYSLELNQFGDESDDEFAMRH
CL6d    LTYGKSYESHE-EEKRFSIWLDNREKINRHNKKFEVGSLSYSLELNKFGDESDDEFAMRH
CL6e    ---------------------------------------------------------MRH

                                       ⬇                         ▽
CL1     GHRADLK-------TESRGSAFIPNACPF-ELPERVDWRDKGLVTPVKNQGQCGSCWAFS
CL2     VSKKHPHLLARQRFGQVFNKTELKARIGKLGLPTNVDWRHSGFVNPPKRQGECRSSYAFS
CL3     GYKAVMKK----HKDAPKGSTYISPSVDF-KLPTDVDWRNDGAVTDVKNQGQCGSCWAFS
CL4     KSKQTRKPKEHTINRLSLNVQELERKLGRRELPKSVDWRTFGVINNVKNQGRCGSCYAFA
CL5     GHRADML-------VVRSGSTYLPHLIDVSNLPDHVDWRTKGAVTDVKDQGQCGSCWAFS
CL6a    KKNMGKKVKEQKLNRVSLNVQQLEKKLGRRDLPQSIDWRNFGVVNRVKDEGNCGSCYAFA
CL6b    ------------------------------------------------------------
CL6c    KKNTGKKQKEQNYNRVSLNVQQLERKLGRRSLPQSVDWRNFGVINQVKDQGDCGSCYAFA
CL6d    KKNKGNKEKDQNFNRVSLNVQQLERKLGRRNLPQSVDWRNFGVINRIKDQGDCGSCYAFA
CL6e    KKNKGKKGKDQNLNRVSLNVQQLERKLGRRNLPQSVDWRNFGVINRIKDQGDCGSCYAFA
                                       ⬆

                                                    67
CL1     TTGSLEGQHFRKTGKLLSLSEQQLVDCSSAFGNHGCNGGLFDFAFKYVQDSGGITTEDLY
CL2     TVASIETYWAKKSGQLLQLSEQQLMDCATSQKMDPCKDNWPNYAFDYII-LNGLTTEDNY
CL3     TTGSLEGQHFRKTGNLVSLSEQQLVDCSSSYGNMGCNGGLMDNAFAYIKATNGIDYEDKY
CL4     TVCTIESHYAIKTSQLLNLSEQQIVDCAIDEGDEGCNGGLMDFSYDYVL-IRGLTKAQNY
CL5     TTGSLEGQHFRKTGKLVSLSEQQLTDCSSSYGNQGCNGGLVDQAFQYVRDCGGLQSENSY
CL6a    TVCTIESHYAIKTGQLLKLSEQQIVDCAGEEGDEACDGGLPDFTYDYVF-FRGLTTEKNY
CL6b    -------------------SEQQICSTCAIDEGDDACDGGLPDFSYDYVL-IRGLTTEKDY
CL6c    TVCTIESHYAIKTSHLLRLSEQQIVDCAIDEGDDACDGGLPDFSYDYVL-IRGL------
CL6d    TVCTIESHYAIKTSHLLRLSEQQIVDCAIDEGDDACDGGLPDYSYDYVL-IRGLTTEKNY
CL6e    TVCTIESHYAIKTSHLLRLSEQQIVDCAIDEGDDACDGGLPDYSYDYVL-IRGLTTEKDY
                             .:*    *:        *...   : :: *:     *:

                                                           133
CL1     PYVSGVIQKAHDVCSYNPDMCKATCTGWVDIPSKDSKALMYAVATIGPISIAINAMGPGF
CL2     SYRAKKG-----QCKEIQIKDVVWIYNYVDLPANDEHAMQLTLALLGPLSVVLEAA-PDL
CL3     PYVSGDTGSAEDTCYFKEEDIGAVDTGYVDIPTEDEAALQEAVANVGPVSVAINAGRADF
CL4     PYKAKSG-----VCKDDKIEPAVHIHSYVDLPSNDEYALKLSLALLGPVAVAIDAE-NDF
CL5     PYISGATSQA-ASCNFDPNKIAATCTGFVDLPEGDEAALMHALATTGPVSIAINAEPADF
CL6a    PYKEKMG-----ICMDHKYKPAVHIHNYVDLPVEDEYALKLSVALLGPVAVGIDAD-NNF
CL6b    PYKDGMT-----ICKDDKFKPAVHIHSYVDLPAQDEYAMKLSVALMGPVAVGIDAE-NTF
CL6c    ------------------------------------------------------------
CL6d    PYKEGMT-----ICKDDKFK----------------------------------------
CL6e    PYKEGMT-----ICKDDKFKPAVHIHSYVDLPAQDEYAMKLSVALMGPVAVGIDAE-NTF

                          157▽                      ▽
CL1     MQYKSGIYNPPSCPGDFSDLDHAVLLVGYGTQ--NGLNYWIVKN-SWSEKWGENGYVRIC
CL2     QFYAGGVYNSVQCHDQEETFDHAMNIVGYGTDEQSGQNYWLVRN-SWGESWGENGYIRIA
CL3     MMYKQGIYKPDECPGQMNDLDHGVLVVGYGSE--NGQDYWIVKN-SWGPDWGESGYIRMA
CL4     RHYKKGVFNSTICDDDPETLDHALTVIGYGTDRVTGQDYWLVRN-SWGSSWGENGYIRIA
CL5     MSYRQGIYENPMARGDRSNLDHAVLCIGYGSE--NGKKYWLVKN-SWSGAWGEQGYIRIA
CL6a    KFYKGGVFNSTMCDDDLATLDHAAVVIGYGNDKITGQDYWLVRN-SWGASWGESGYVRYA
CL6b    RFYKGGVFNSTMCDDDPSTLDHAVVVIGYGTDEVSGQDYWLVRN-SWSASWGESGYMRYA
CL6c    ------------------------------------------------------------
CL6d    ------------------------------------------------------------
CL6e    RFYKGGVFNSTMCHDDPSALDHAVVVIGYGTDEVSGQDYWLVRNNSCGTSWGESGYIRYA

                      205
CL1     RD-GRNLCGVATCASYPLV
CL2     RTTPNNLCGIAERPSYPLV
CL3     RN-SGNLCGIATAASYPLV
CL4     RTNPANLCGITDAASFPLV
CL5     RD-DRNICGVTTLASYPLV
CL6a    RTNPHNLCGITDSASFPLV
CL6b    RTNPHNLCGITDMASFPLV
CL6c    -------------------
CL6d    -------------------
CL6e    RTNPQNLCGITDDASFPLV
```
